# A novel framework unveiling the importance of heterogeneous selection and drift on the community structure of symbiotic microbial indicator taxa across altitudinal gradients in amphibians

**DOI:** 10.1128/spectrum.04192-23

**Published:** 2025-01-08

**Authors:** Jin Zhou, Zhidong Liu, Sishuo Wang, Jing Li, Lin Zhang, Ziyan Liao

**Affiliations:** 1 Chengdu Institute of Biology, Chinese Academy of Sciences, Chengdu, China; 2 Key Laboratory of Bio-Resources and Eco-Environment of Ministry of Education, College of Life Sciences, Sichuan University, Chengdu, Sichuan, China; 3 University of Chinese Academy of Sciences, Beijing, China; 4 Department of Microbiology, The Chinese University of Hong Kong, Shatin, Hong Kong SAR, China; Brigham Young University, Provo, Utah, USA

**Keywords:** community assemblage, indicator taxa analysis, natural selection, neutral theory, turnover, taxon-specific effect

## Abstract

Existing analytical frameworks for community assembly have a noticeable knowledge gap, lacking a comprehensive assessment of the relative contributions of individual or grouped microbial distinct sampling units (DSUs) and distinct taxonomic units (DTUs) to each mechanism. Here, we propose a comprehensive framework for identifying DTUs/DSUs that remarkably contribute to the various mechanisms sustaining microbial community structure. Amphibian symbiotic microbes along an altitudinal gradient from Sichuan Province, China, were employed to examine the proposed statistical framework. In different altitude groups, we found that heterogeneous selection governed the community structure of symbiotic microbes across DSUs, while stochastic processes tended to increase with altitude. For DTUs at phylum and family levels, drift emerged as the dominant mechanism driving the community structure in the most symbiotic microbial taxa, while heterogeneous selection governs the most dominant or indicator taxa. Notably, the relative contribution of heterogeneous selection was significantly positively correlated with the relative abundance and niche breadth of taxa, and negatively correlated with drift. We also detected that community assembly processes remarkably regulate the structure of symbiotic microbial communities and their correlation with environmental variables. Altogether, our modeling framework is a robust and valuable tool that further enlarges our insight into microbiota community assembly.

**IMPORTANCE:**

Distinguishing the drivers regulating microbial community assembly is essential in microbial ecology. We propose a novel modeling framework to partition the relative contributions of each individual or group of microbial DSUs and DTUs into different underpinning mechanisms. An empirical study on amphibian symbiotic microbes notably enlarges insight into community assembly patterns in the herpetological symbiotic ecosystem and demonstrates that the proposed statistical framework is an informative and sturdy tool to quantify microbial assembly processes at both levels of DSUs and DTUs. More importantly, our proposed modeling framework can provide in-depth insights into microbiota community assembly within the intricate tripartite host-environment-microbe relationship.

## INTRODUCTION

Untangling community assembly patterns is a central issue in microbial community ecology (1). Microbial community assembly is operated by simultaneously deterministic processes based on the niche theory and stochastic processes rooted in the neutral theory. Deterministic processes refer to the environmental filtering (e.g., PH) and interspecies interactions (e.g., predation) that shift the microbial community’s structure and composition, which are further categorized into two types: heterogeneous (or variable) selection which promotes community-level microbial divergence, and homogeneous selection which prevents community-level microbial divergence (2–4). Conversely, stochastic processes assume that all microbial taxa are ecologically equivalent, and community structure is mediated by birth, death, dispersal, speciation, and extinction, which primarily include dispersal limitation, homogeneous dispersal, and drift (see Supplementary file for terms and definitions used for community assembly models) (3, 5, 6). Many studies have attempted to evaluate the relative strength of deterministic processes versus stochastic processes using variation partitioning techniques derived from multivariate statistical methods (e.g., redundancy analysis, RDA) (7, 8). An ecology-worthy two-step analytical framework (3, 9) has been established to distinguish the relative influence of various mechanisms in shaping the microbial community assembly. A two-step analytical pipeline is elegantly designed: the first step aims to distinguish the relative influences of heterogeneous and homogenizing evolutionary selection by conducting randomization tests on the phylogenetic beta diversity patterns of microbial communities, while the second step seeks to disentangle the relative contributions of dispersal limitation and homogenizing dispersal.

Several important but unsolved questions that are closely associated with Stegen et al.’s (3) modeling framework persist, and we will concentrate on two of them in this study. First, which particular taxon or sets of taxa (referring to different taxonomic units [DTUs] of microbial taxa, such as phylum, class, order, family, genus, and OTUs [or ASVs] of microbial taxonomy) have played a notable role in the processes of selection, dispersal, and drift within a specific microbial community? Second, for the geographic units or host species body parts where microbes have been sampled, which individuals or groups of sampling units (referring to individuals or groups of different sampling units [DSUs], such as sample individuals along an elevation gradient and corresponding sample grouping of low-middle-high elevation) have been principally structured by selection, dispersal, or drift? (see Supplementary file for terms and definitions). In this study, we developed a novel modeling framework based on Stegen et al. (3) for identifying the relative contributions of individuals or groups of DTUs/DSUs in the community assembly. Identification of the relative contributions of individuals or groups of microbial DTUs/DSUs has practical implications. For example, microbial taxa in polluted environments are detected under strong, differentiating selection, and might be valuable for ecological restoration and environmental management. Furthermore, taxa subject to strong dispersal limitation could be valuable for classifying and mapping polluted landscapes.

Animal symbiotic microbiota has become a recurrent research topic in recent years (10, 11), yet little is known about the community assembly rules structuring symbiotic microbiota, particularly on poikilothermal animals like amphibians. Symbiotic microbes may be a suitable model for evaluating the impacts of different community assembly rules on host-associated microbial diversity and community structure. They are subject to selection from both external abiotic (outside the body) and internal biotic (inside the body) influences. Since poikilotherms are heavily influenced by the temperature change of surrounding abiotic environments, it is expected that heterogeneous selection should have a great impact on the symbiotic microbial community. Moreover, the symbiotic microbiota is subject to dispersal filtering if sampled from distinct locations. It would be fascinating to know which microbial taxa in the guts or skin of poikilothermal animals are strongly tied to selection and those associated with dispersal.

In this study, we tested our proposed modeling framework by field sampling amphibian symbiotic microbes along an altitudinal gradient in Sichuan Province, China. The amphibian was selected on the grounds of its wide distribution across different altitudinal gradients and its sensitivity to environmental change (12, 13), which contributes to our better deciphering of the relative importance of selection and dispersal in structuring the symbiotic microbiota of amphibians. Hence, this study aimed to (i) explore the spatial variation of the relative contribution of DSUs and DTUs in regulating symbiotic microbial community structure along an altitudinal gradient, (ii) detect how different community assembly processes affect the community structure of symbiotic microbes, such as relative abundance and ecological niche width, and (iii) disentangle how five community assembly processes regulate the relationship between the relative abundance of symbiotic microbes and environmental factors.

## MATERIALS AND METHODS

### Sampling and sequencing

**Fig 1:**
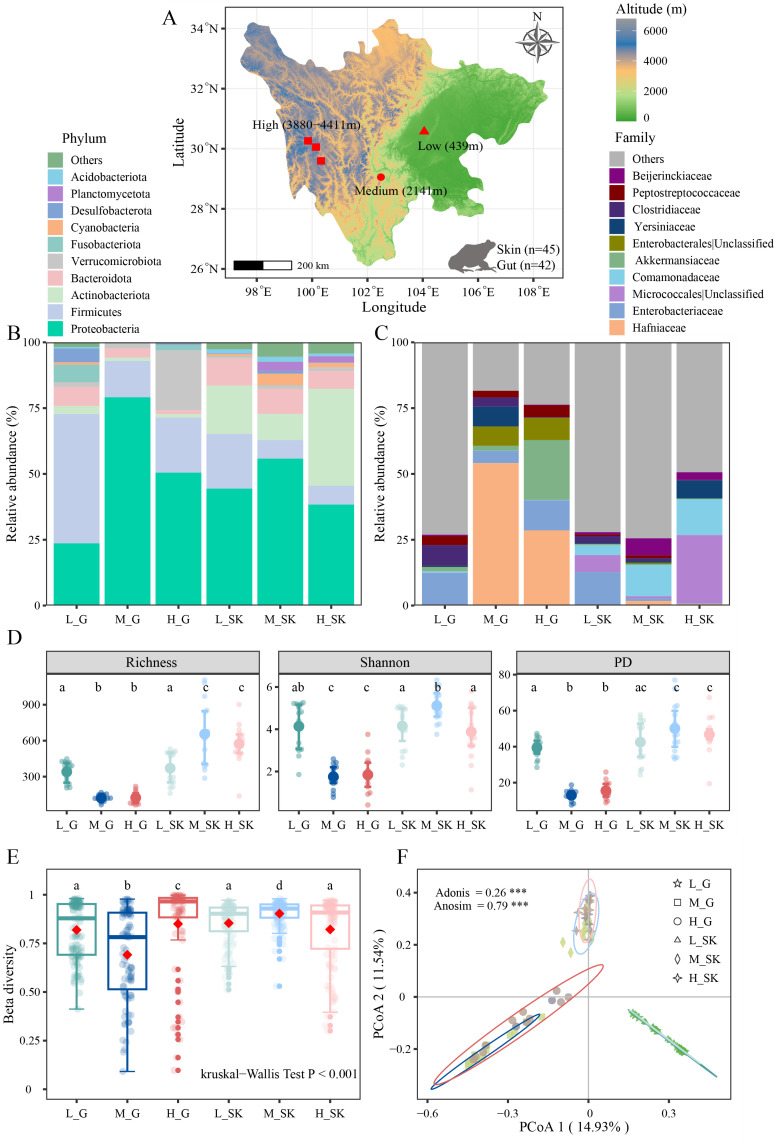
Basic analysis of the structure of the symbiotic microbial community. (**A**) Distribution of sample points for different elevation gradients, *n* represents the number of samples. Free China GIS Map Files were downloaded from: https://simplemaps.com/gis/country/cn#admin1 (License: Creative Commons Attribution 4.0). Stacked map of the top 10 phyla (**B**) and families (**C**) in relative abundance. (**D**) Comparison of α-diversity (richness, Shannon index, and phylogenetic diversity: PD) along different altitudinal gradients. (**E**) Comparison of β-diversity (dissimilarities) based on the Bray–Curtis distance. The red points represent the mean values. The darker colors of green, blue, and brown represent the low-, medium-, and high-altitude grouping of gut microbes, while the corresponding lighter colors represent the low-, medium-, and high-altitude grouping of skin microbes. (**F**). PCoA analysis built in the Bray–Curtis distance. L_G, M_G, and H_G represent gut microbes at low, mid, and high altitudes, while L_SK, M_SK, and H_SK represent skin microbes at low, mid, and high altitudes, respectively. Significant differences (*P* < 0.05) between groups are represented by different superscript letters. Differences are denoted as follows: ∗*P* < 0.05; ∗∗*P* < 0.01; ∗∗∗*P* < 0.001.

A total of 87 amphibian symbiotic microbial samples (Fig. 1A; Table S1) were collected from June to August 2021 along an altitudinal gradient in Sichuan Province, China, comprising the low-, medium-, and high-altitude groups (Fig. 1A). At each sampling site, we captured adult amphibians by wearing sterile gloves along about 500 m length of the river or pond. Following, we quickly rinsed the skin with sterile water to eliminate the potential transient bacteria and then swabbed the amphibian’s skin three times with sterile cotton swabs to collect microbial samples from the skin (14). After being transported to the laboratory and the frog was euthanized and dissected, microbial content from the gut was collected in an aseptic centrifuge tube of 2 mL. Samples were dried and crushed using liquid nitrogen and stored at −80°C until DNA was extracted from them. Experimental approval was obtained from the Animal Ethical and Welfare Committee of Chengdu Institute of Biology, Chinese Academy of Sciences (permit no. CIBDWLL2022008).

The metagenomic DNA was extracted from 250 mg contents of each sample using the MN NucleoSpin 96 Soil kit (MACHEREY-NAGEL) following the manufacturer’s protocol and was then verified by 1.0% agarose gel electrophoresis and amplified the V3-V4 hypervariable regions of the 16S rRNA gene using the two universal bacterial primers 338F (5′ACTCCTACGGGAGGCAGCA-3′) and 806R (5′-GGACTACHVGGGTWTCTAAT-3′). Sequencing was performed by Biomarker Technologies. Finally, an Illumina Nova platform (Nova6000 pe250) was used to sequence the amplicon libraries with 250 bp paired-end reads. Here, all raw reads were processed *via* the QIIME2 (v2021.2) software package (15). The amplicon sequence variants (ASVs) were produced through the DADA2 pipeline (16). Subsequently, all samples were rarefied to the identical sequencing depth (28,628 reads per sample). To mitigate the effects of sequencing errors and rare taxa, we further filtered the feature tables with QIMME2 feature-table filter-features (p-min-samples 2 --p-min-frequency 2) (17). The Silva v138 database and the Naive Bayes classifier were applied for the ASVs taxonomy assignment (18). Any sequences identified as chloroplasts derived or mitochondria derived were filtered out resulting in 8,861 ASVs for downstream analysis.

### A two-step analysis for identifying the strength of different mechanisms

We implemented Stegen’s two-step algorithm for identifying the relative strength of selection, dispersal limitation, and random drift in structuring the microbial community structure. To be specific, pairwise phylogenetic turnover was characterized using the abundance-weighted beta-mean-nearest taxon distance ($\beta_{MNTD}$) (3, 9, 19), which is computed for a pair of microbial communities as follows:


$$\beta_{MNTD}=\frac{1}{2}[\sum_{i_{k}=1}^{n_{k}}f_{i_{k}}\min\left(d_{i_{k}j_{m}}\right)+\sum_{i_{m}=1}^{n_{m}}f_{i_{m}}\min\left(d_{i_{m}j_{k}}\right)]$$

where $f_{i_{k}}$ is the relative abundance of species *i* from community *k*, and $n_{k}$ is the number of species in community *k*. $\min\left(d_{i_{k}j_{m}}\right)$ is the minimal phylogenetic distance between species *i* from community *k* to all species *j* from another compared community *m*. The definitions of $f_{i_{m}}$, $n_{m}$, and $\min\left(d_{i_{m}j_{k}}\right)$ follow a similar logic.

In practice, because of the involvement of randomization (i.e., randomly permuting the tip labels of the phylogenetic tree), we used the standardized effective size of $\beta_{MNTD}$ (${SES.\beta }_{MNTD}$), which is simply the difference between the observed value and the mean of null-model values derived from randomization, divided by the associated standard deviation. If variable or heterogeneous selection is operating on a pair of microbe communities, the associated ${SES.\beta }_{MNTD}$ for the pair of communities should be significantly higher than the null expectations. By contrast, if the homogenizing selection is operating on a pair of microbe communities, the associated ${SES.\beta }_{MNTD}$ for the pair of communities should be significantly lower than the null expectations.

Finally, the remaining pairs of microbial communities without significant ${SES.\beta }_{MNTD}$ values were subject to the second stage of analyses, which is focused on taxonomic turnover. The Raup–Crick metric was applied to infer the taxonomic turnover (3, 4). Specifically, the calculated metric is called the Raup–Crick metric for the Bray–Curtis dissimilarity (${RC}_{bray}$), and randomization is used to generate a null distribution for testing the significance of the metric (3, 9, 20, 21). To be more specific, if the observed ${RC}_{bray}$ value has a probability of being larger than 95% of the values derived from those randomized null models, that is, RC_bray_ > 0.95, this pair of sites was considered to be subject to dispersal limitation. By contrast, if RC_bray_ < 0.95, it is believed that this pair of sites is subject to homogenizing dispersal. The randomization procedure here complies with the following rules: (i) taxonomic richness in each sampling site is fixed (20) and (ii) the probability of a species being assigned to a site is proportional to its range size (i.e., the number of sites occupied), while the probability of assigning an individual into a species in a site was proportional to the relative abundance of the species across all sites (3, 9). Finally, after the pairs of sites being subject to significant dispersal limitation were identified, the remaining pairs of sites were classified as those subject to random drift. The *comdistnt* function in the R package “picante” calculates the ${SES.\beta }_{MNTD}$, and the ${RC}_{bray}$ calculation code is obtained from Chase et al. (20).

### Identifying relative contributions of individuals or groups of DTUs

Here, the ASVs were used to represent DTUs for subsequent analyses. To identify the relative contributions of each microbial ASV or groups of ASVs (i.e., family and phylum) to selection, dispersal, and drift, we used a taxon-specific or group-specific removal procedure. Briefly, when implementing randomization tests in the first and second steps of Stegen et al. (3)’s analytical pipeline, for each randomization, we removed the targeted ASV or the ASVs group (refer to higher levels of microbial taxonomy, such as phylum and family) from the community and recalculated the associated metrics, including the standardized $\beta_{MNTD}$ and${RC}_{bray}$.

To identify the contribution of each taxon or taxonomic group to each mechanism, we followed two basic criteria. First, before the taxon is removed, the metrics ${SES.\beta }_{MNTD}$ (or ${RC}_{bray}$) could be significant; but after the taxon is removed, these metrics become nonsignificant. Second, before the taxon is removed, the metrics ${SES.\beta }_{MNTD}$ (or ${RC}_{bray}$) could be nonsignificant; but after the taxon is removed, these metrics become significant.

The following five decision rules that implement the above basic criteria were then used to diagnose the importance of each taxon and its relative role involved in different forms of selection, dispersal, and drift:

1. A taxon *i* is believed to have a remarkable contribution to the variable or heterogeneous selection process for structuring microbial community structure if this condition is satisfied simultaneously:

$$\left\{ \begin{array}{l} 2<SES.\beta_{MNTD}\\ SES.\beta_{MNTD}\left(-i\right)<2 \end{array}\right.$$

${SES.\beta }_{MNTD}$ is the original standardized value calculated from all taxa, and ${SES.\beta }_{MNTD}\left(-i\right)$ is the new standardized value for which the focused taxon is removed when computing the phylogenetic turnover metric.

- A taxon *i* is believed to have a remarkable contribution to the homogeneous selection process for structuring microbial community structure if the following conditions are satisfied simultaneously:

$$\left\{ \begin{array}{l} -2>SES.\beta_{MNTD}\;\\ SES.\beta_{MNTD}\left(-i\right)>-2 \end{array}\right.$$

- A taxon *i* is believed to have a remarkable contribution to the homogenizing dispersal process for structuring microbial community structure if these conditions are satisfied simultaneously:

$$\left\{ \begin{array}{l} -2\leq SES.\beta_{MNTD}\leq 2\\ -2\leq SES.\beta_{MNTD}\left(-i\right)\leq 2\\ 0.95<RC_{bray}\\ RC_{bray}\left(-i\right)\leq 0.95 \end{array}\right.$$

${RC}_{bray}$ is the original taxonomic turnover Raup–Crick metric calculated from all taxa, and ${RC}_{bray}\left(-i\right)$ is the new Raup–Crick metric for which the focused taxon is removed when computing the taxonomic turnover metric.

- A taxon *i* is believed to have a remarkable contribution to the dispersal limitation process for structuring microbial community structure if these conditions are satisfied simultaneously:
$$\left\{ \begin{array}{l} -2\leq SES.\beta_{MNTD}\leq 2\\ -2\leq SES.\beta_{MNTD}\left(-i\right)\leq 2\\ -0.95>RC_{bray}\\ RC_{bray}\left(-i\right)\geq -0.95 \end{array}\right.$$
- Finally, if none of the above four criteria are met, then the taxon is believed to contribute to drift.

**Fig 2:**
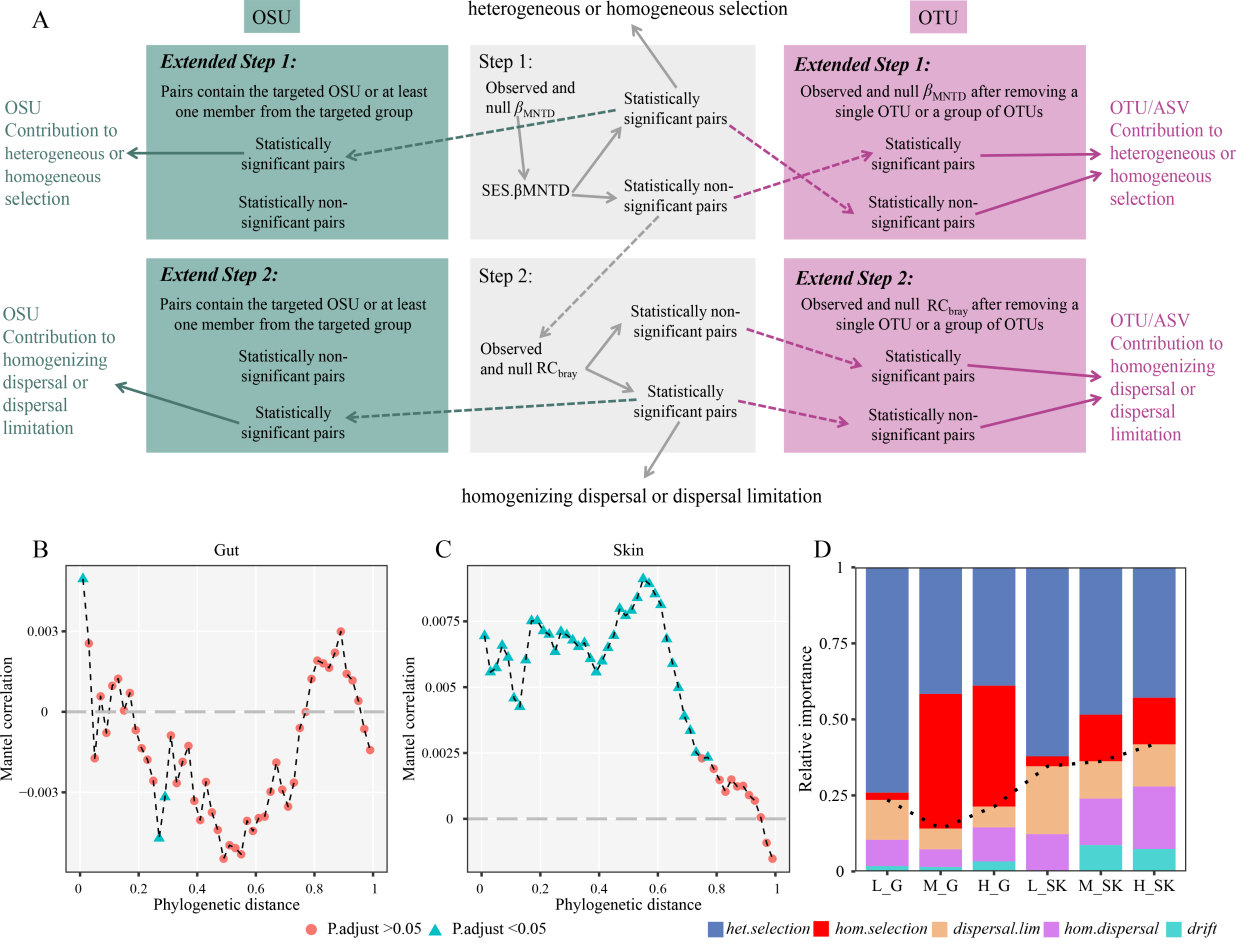
(**A**) A schematic flowchart for showing the proposed modeling framework for identifying the contribution of individuals or groups of microbial distinct taxonomic units (DTUs, e.g., ASVs, family and phylum) and sampling units (DSUs, e.g., sample individual or sample grouping) on each of the underlying microbial community assembly rules. The proposed extended analyses are illustrated in the palevioletred on the right (DTUs) and the dark cyan on the left (DSUs). Detection of phylogenetic signals in gut (**B**) and skin (**C**) microorganisms along an altitudinal gradient. (**D**) The relative contribution of different community assembly processes along an altitudinal gradient based on the contribution analysis of DSUs. The black line represents the trend of stochasticity along the elevation gradient.

The above five decision rules are exclusively discussed in the context of a specific ASV. However, the removal algorithm for a group of ASVs followed the decision rules as long as the index i is used to depict a group of ASVs. Furthermore, in our proposed protocol of identifying the relative contributions of different microbial ASV individuals or groups for different mechanisms (i.e., selection, dispersal, and drift), to avoid the influence of random fluctuation, the taxon-specific removal algorithm is implemented simultaneously during the first and second steps when Stegen et al.’s analytical framework is implemented. A schematic flowchart of implementing the proposed ASV-specific or ASV group-specific removal algorithm is presented in Fig. 2A.

### Identifying relative contributions of individuals or groups of DSUs

To identify the relative contributions of individuals or groups of DSUs in the maintenance of microbial diversity and community structure, we used a strategy different from the removal algorithm used for identifying the contributions of individuals or groups of DTUs. A schematic flowchart of implementing the proposed DSU-specific or DSU group-specific contribution analysis algorithm is presented in Fig. 2A.

To be more specific, since Stegen’s analytical framework is established based on the comparison of beta diversity between each pair of sampling units (3, 9), during the first and second steps of the implementation of Stegen’s method, we counted the number of pairs of sites that presented significant results (e.g., significant ${SES.\beta }_{MNTD}$ or ${RC}_{bray}$) and contained at least one of the targeted sampling units. For example, for a hypothetical microbial community collected from four DSUs (suppose their IDs are A, B, C, and D), there are 4×3/2=6 pairs of sampling units for analyses when implementing Stegen’s framework. Suppose that we are interested in the joint contribution of two sites, A and B, in different maintenance mechanisms; we counted pairs of sites where each or both sites A and B presented, including pairs AB, AC, AD, BC, and BD. We then counted the number of pairs that presented significant ${SES.\beta }_{MNTD}$ or ${RC}_{bray}$, divided by the total number of pairs containing the targeted single DSU or at least one of the targeted DSUs in a group (for the hypothetical case here, the group contains A and B and five pairs of sites containing at least one of the group member sites), to identify the relative contribution of each single DSU or a group of DSUs in different maintenance mechanisms of community structure.

### Bioinformatics analysis

All data analysis and visualization were accomplished with R software (v4.2.2) (22). All values were presented as mean ± standard deviation. To examine the community structural and diversity variation of symbiotic microbes along the altitudinal gradient, we first evaluated their compositional differences at the phylum and family levels and then calculated richness, Shannon, and phylogenetic diversity (PD) to estimate α-diversity with the “vegan” package (23, 24). Next, the Bray-Curtis distance was applied to determine the spatial variation in β-diversity across the altitudinal gradient. ANOSIM and PERMANOVA analyses (number of permutations: 999) based on Bray-Curtis distances were employed to examine the differences in β-diversity. Principal coordinate analysis (PCoA) was used to calculate and visualize the results. The Wilcoxon rank-sum test and FDR (false discovery rate)-adjusted *P* values were applied to compare the α- and β-diversity differences at distinct altitudinal gradients, while the Kruskal–Wallis test was applied to compare the overall differences in the median between the different groups.

Importantly, for examining the relative contribution of different maintenance mechanisms on microbial diversity and community structure in sampling sites and taxonomic units (Fig. 2A), we developed *ad hoc* R codes for the calculation (see the supporting information). Prior to conducting $\beta_{MNTD}$, it is essential to test for the significance of the phylogenetic signal, which determines whether phylogenetic turnover can be reliably used for ecological inference within the system (3, 4). First, we use Mantel correlograms to test the phylogenetic signals (999 randomizations) of the symbiotic microbial communities at different altitudinal gradients, with the calculation using the *mantel.correlog* function of “vegan” package (23, 25). Second, we analyzed the relative contribution of DTUs and DSUs in sustaining symbiotic microbial community assembly with randomization 500 times at different altitudinal gradients, respectively. Here, for the DTUs, we mainly focus on contribution analysis at the phylum and family levels. Furthermore, we also computed the niche breadth of phyla and families of symbiotic microbes, and classified them into three groups: specialist, neutral, and generalist, *via* the quasiswap permutation algorithm (simulating 1000 permutations) built on their occurrences (2, 26). These analyses were implemented using the *spec.gen* function in the “EcolUtils” package (27). Generally, taxa with large niche breadth values are more abundant, distributed evenly, and more competitive (28). In addition, generalists have a broader niche breadth than specialists and may be more competitive (29). Linear discriminant analysis (LDA) effect size (LEfSe) method was applied to decipher differences in the community structure of distinct altitudinal gradient groups and to identify the corresponding indicator species (30). Only taxa with *P* < 0.05 and LDA score >2 were present. To detect variations in the adaptability of skin and gut microbes to different altitudinal gradients, we compared their differences in composition, niche breadth, and community assembly processes based on the subtraction of their different properties. The ordinary least-squares (OLS) regressions were employed to assess the relative abundance or niche breadth at the phylum and family levels in relationship to the different community assembly processes, respectively. More importantly, we also examined the correlation of differences in distinct community assembly processes of gut and skin microbes with differences in relative abundance or niche breadth, to further determine whether these differences were caused by differences in distinct community assembly processes.

To weigh the influence of environmental factors on the symbiotic microbial community structure, we collected PH, WT (water temperature), AT (air temperature), and AH (air humidity) for each sample site, as well as extracting the 30 s resolution historical climate data (including 19 bioclimatic variables and elevation, Table S2) based on the coordinates of each sample from the WorldClim database (https://www.worldclim.org/). The PH, WT, AT, and AH were measured as previously described (14). In addition, we used a Random Forest model (RF) to assess the importance of these variables on symbiotic microbial richness at different altitudinal gradients using “randomForest” and “rfPermute” packages (31, 32). Larger MSE% (mean squared error) values hinted that the variable was more important. For reducing the multicollinearity of environmental variables, based on the RF model, six representative and significantly important factors were picked for follow-up analysis, comprising WT, PH, lon (longitude), elev (elevation), bio_4 (temperature seasonality), and bio_13 (precipitation of wettest month). In addition, Spearman’s correlation analysis was conducted to estimate the relationship between the relative abundance of symbiotic microbes and environmental factors at the phylum and family levels, respectively. Also, we estimated the relationships between different community assembly processes and the correlations derived from relative abundance and environmental factors, which can provide further insight into the relative importance of ecological processes in regulating the impact of environmental factors on the structure of symbiotic microbial communities.

Multiple regression on matrices (MRMs) was used to explore the extent to which elevation, host species, and habitat types (Table S1) affected skin and gut microbial alpha and beta diversity (33, 34). The α-diversity was represented using Shannon diversity and converted to Euclidean distance, while β-diversity was measured using Bray–Curtis distance. Species and habitat types data were converted to a Gower distance. The *stdize* function of the “MuMIn” package was used to normalize the matrices of the different independent variables to ensure their comparability and the “ecodist” package was used to perform the MRMs (1,000 random permutations).

## RESULTS

### Spatial variation in symbiotic microbial community structure at different altitudinal gradients

Profoundly divergent in the composition of amphibian symbiotic microbes were detected across the altitudinal gradient (Fig. 1A and B; Fig. S2). At the phylum level, Proteobacteria and Firmicutes were the common dominant phylum in both the gut and skin, but Proteobacteria was significantly higher in the mid-altitude group than in the low- and high-altitude groups (Fig. 1B). In addition, Actinobacteriota was significantly higher in the skin than in the gut. Notably, Verrucomicrobiota presented a significant enrichment in the gut of the high-altitude group (H_G), yet Desulfobacterota showed significant enrichment in the low-altitude group (Fig. 1A; Fig. S2A). At the family level, there were striking differences between the dominant families of gut and skin, with Hafniaceae and Akkermansiaceae being the dominant families of gut microbes, while Unclassified of Micrococcales order and Comamonadaceae were the dominant families of skin microbes. Hafniaceae were significantly enriched in the mid- and high-altitude groups of the gut (M_G and H_G), Akkermansiaceae in the high-altitude group (H_G), and Unclassified of Micrococcales in the high-altitude group (H_SK) of the skin (Fig. 1C; Fig. S2B). Besides, Yersiniaceae and Beijerinckiaceae show marked enrichment in the mid-elevation group (M_G and M_SK).

The α-diversity of skin microbes was generally higher than the gut microbes at different altitudinal gradients, and it exhibited an opposite trend from low to high altitude (Fig. 1D; Table S3). The α-diversity of gut microbes showed the following trend: L_G (Richness: 340.13 ± 89.36, Shannon: 4.14 ± 1.16, PD: 39.41 ± 5.65) >H_G (Richness: 124.54 ± 51.16, Shannon: 1.85 ± 0.90, PD: 15.51 ± 4.98) >M_G (Richness: 122.36 ± 27.99, Shannon: 1.75 ± 0.58, PD: 12.99 ± 3.05), whereas the skin microbes presented an opposite trend: M_SK (Richness: 654.80 ± 266.89, Shannon: 5.11 ± 0.78, PD: 50.15 ± 13.83) >H_SK (Richness: 573.53 ± 168.38, Shannon: 3.88 ± 1.29, PD: 46.74 ± 10.26) >L_SK (Richness: 370.27 ± 134.68, Shannon: 4.15 ± 0.98, PD: 42.47 ± 11.70) (Fig. 1D). However, we found that the β-diversity of gut microbes was remarkably higher in high-altitude group (H_G, 0.85 ± 0.23) than in the low- (L_G, 0.82 ± 0.15) and mid-altitude group (M_G, 0.69 ± 0.24) (Fig. 1E; Table S4), and the mid-altitude group had the lowest beta diversity. Conversely, for skin microbes, the high-altitude group (H_SK, 0.82 ± 0.18) had the lowest β-diversity, while the mid-altitude group (M_SK, 0.90 ± 0.07) did have the highest β-diversity, implicating that the skin microbes had a higher dispersion or turnover in mid-altitude habitats. Furthermore, PCoA analysis validated the significant differences in the structure of the symbiotic microbial community at different altitudinal gradients (Adonis: R = 0.26, *P* < 0.001; Anosim: R = 0.79, *P* < 0.001; Fig. 1F).

### Relative contribution of DSUs/DTUs in manipulating symbiotic microbial community assembly along altitudinal gradients

Significant phylogenetic signals in relatively short phylogenetic distances were found in symbiotic microbes across the altitudinal gradient, and skin microbes had stronger phylogenetic signals than gut microbes (Fig. 2B and C), which implies that it is reasonable for our model to use phylogenetic turnover analysis to infer underlying ecological processes (25). At the level of DSUs, heterogeneous selection was the dominated symbiotic microbial community assembly, but their relative importance tended to decrease with rising altitude (Fig. 2D). In contrast to low-altitude group, the relative importance of homogeneous selection and homogenizing dispersal was higher in the mid- and high-altitude groups, while dispersal limitation showed the opposite trend (Fig. 2D). Furthermore, the stochasticity of microbial community assembly was markedly higher in skin than in the gut, likely attributed to the greater sensitivity and uncertainty of host skin to environmental shifts.

**Fig 3:**
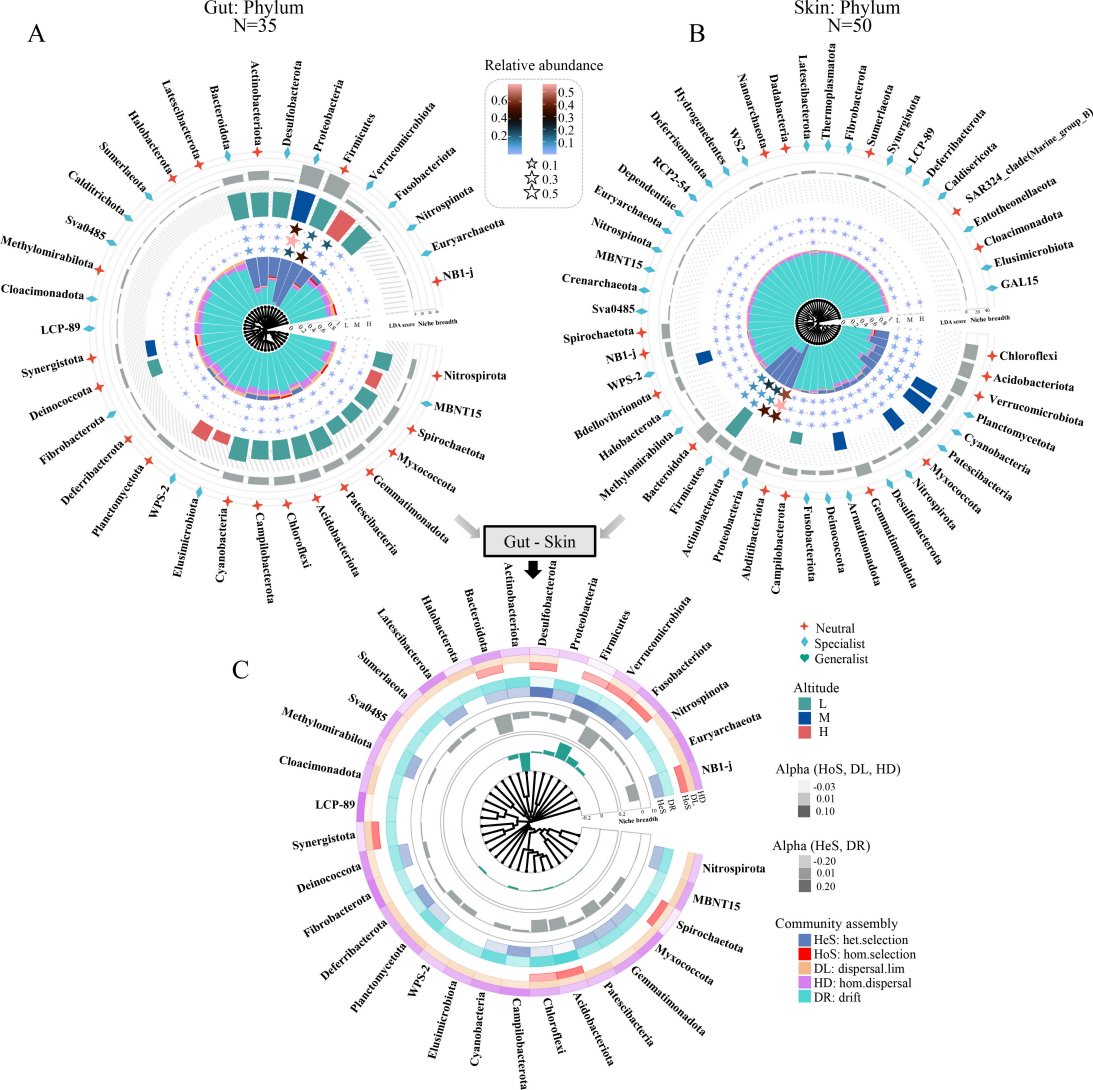
Distribution patterns in gut (**A**) and skin (**B**) microbial community structure and assembly at the phylum level along an altitudinal gradient. The innermost layer represents a clustering tree based on the Bray–Curtis distance. The penultimate layer suggests a distribution of community assembly processes. The pentagrams denote the relative abundance of taxa and the size or color represents the extent of the values (only taxa are shown with value >0). The different colors of the bars correspond to the absolute value of LDA scores of the indicator taxa for the different altitude gradients. The gray bars show the niche breadth of the taxa. The outermost layer implies the classification of different taxa, including neutral, specialist, and generalist. (**C**) Comparison of differences (gut minus skin) in gut and skin microbial community structure (including differences in relative abundance in the penultimate layer and differences in niche breadth in the gray bars) and community assembly (outermost heat map) at the phylum level. hom.selection: homogenizing selection; het.selection: heterogeneous selection; hom.dispersal: homogenizing dispersal; dispersal.lim: dispersal limitation.

For the DSUs at the phylum level (ASV groups), we found that drift processes likely dominated the assembly of most phyla in the gut and skin microbial communities, while dispersal limitation and homogenizing dispersal play a relatively smaller role (Fig. 3A and B). Nonetheless, in gut microbes, the low-altitude indicator phylum Firmicutes, mid-altitude indicator phylum Proteobacteria, and high-altitude indicator phylum Verrucomicrobiota were all primarily driven by heterogeneous selection (Fig. 3A), which might result from these phyla having a wider niche breadth and long-term host selection or environmental filtering. Notably, the heterogeneous selection had no contribution to the other three high-altitude indicator phyla (WPS-2, Elusimicrobiota, and MBNT15), but both dispersal processes played an important role in their community assembly (Fig. 3A). In skin microbes, these dominant phyla are driven by similar mechanisms, but none of them is considered indicator phylum (Fig. 3B). In addition, there are phyla that are not dominant phyla but for which heterogeneous selection also plays a pivotal role, such as Chloroflexi and Acidobacteriota (Fig. 3B). Interestingly, we identified different role-playing of the same species in different symbiotic organs of the host, for example, Firmicutes as a neutral in the gut and a specialist in the skin, and Verrucomicrobiota with opposite transformation patterns, most likely driven by the variability in the different communities assembly processes (Fig. 3A and B). As such, we compared the variability in some properties of gut and skin microbes at the phylum and family levels, including community assembly, niche breadth, and relative abundance (Fig. 3C). The findings showed significant differences in three attributes of the same phylum in both gut and skin microbes (Fig. 3C), suggesting significant partitioning of these taxa in the gut and skin. For instance, in contrast to skin microbes, the relative abundance, niche breadth, and heterogeneous selection of Firmicutes in gut microbes showed a significant increase, while homogenizing dispersal and drift presented a decrease (Fig. 3C).

**Fig 4:**
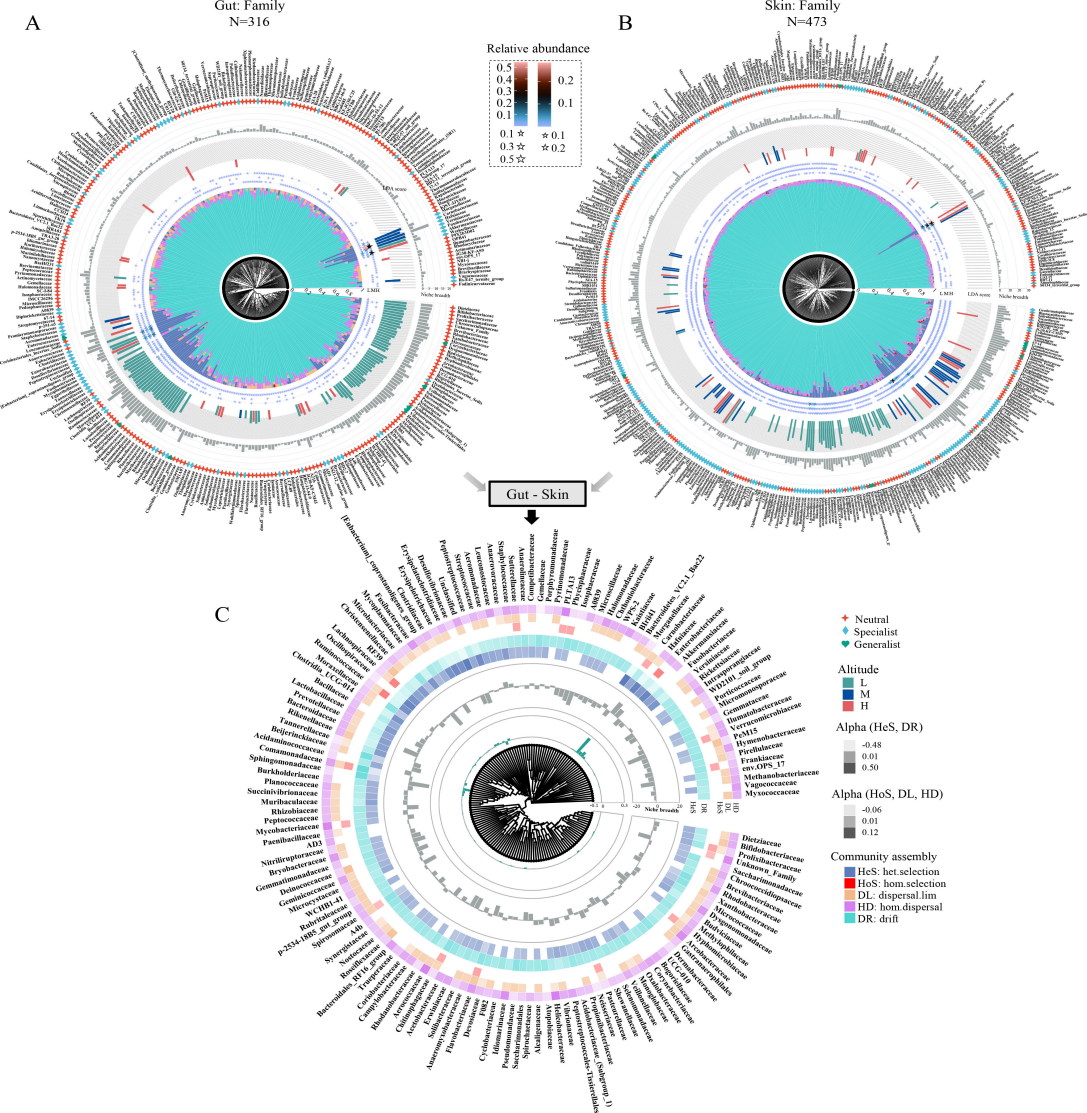
Distribution patterns in gut (**A**) and skin (**B**) microbial community structure and assembly at the family level along an altitudinal gradient. The innermost layer represents a clustering tree based on the Bray–Curtis distance. The penultimate layer indicates a distribution of community assembly processes. The pentagrams denote the relative abundance of taxa and the size or color represents the extent of the values (only taxa are shown with value >0). The different colors of the bars correspond to the absolute value of LDA scores of the indicator taxa for the different altitude gradients. The gray bars show the niche breadth of the taxa. The outermost layer implies the classification of different taxa, including neutral, specialist, and generalist. (**C**) Comparison of differences (gut minus skin) in gut and skin microbial community structure (including differences in relative abundance in the penultimate layer and differences in niche breadth in the gray bars) and community assembly at the family level (only taxa with LDA scores > 0 are shown).

At the family level, heterogeneous selection and drift govern the community assembly of most families of gut and skin microbes, in combination with the contribution of two dispersal processes (Fig. 4A and B). In addition, we also found that most indicator species had a high niche breadth and were accompanied by a high relative contribution of heterogeneous selection (Fig. 4A and B), which may implicate that heterogeneous selection can markedly alter the ecological adaptations of indicator species and thus control their community assembly. For example, in gut microbes (Fig. 4A), some dominant families have a high proportion of heterogeneous selection, as high-altitude indicator family Akkermansiaceae, low-altitude indicator family Fusobacteriaceae and mid-altitude indicator families Yersiniaceae and Hafniaceae, all of which however are specialists. Nevertheless, some of these families have undergone remarkable changes in the properties (e.g., niche breadth, community assembly processes) of their skin microbes, and some are not indicator species (Fig. 4B). Noticeably, besides drift, homogenizing dispersal, and dispersal limitation play critical roles in community assembly of some indicator taxa, while heterogeneous selection does not contribute, as seen in Idiomarinaceae, Methanobacteriaceae and Verrucomicrobiaceae, etc (Fig. 4A and B).

In addition, this variability is more pronounced at the family level compared to the variability in the different properties of gut and skin microbes at the phylum level (Fig. 4C). To examine the relationship between these variabilities and the difference of distinct community processes, we conducted an OLS regression analysis, which showed that heterogeneous selection differences and drift differences were positively correlated with the niche difference and relative abundance difference at the phylum level, whereas at the family level, only significant correlations were found with relative abundance differences (all *P* < 0.05, Fig. S4). Furthermore, dispersal limitation differences also showed remarkable negative correlations with differences in relative abundance and niche (Fig. S7). The above results suggest that these differences of gut and skin microbes are likely caused by the variability of their community assembly processes.

**Fig 5:**
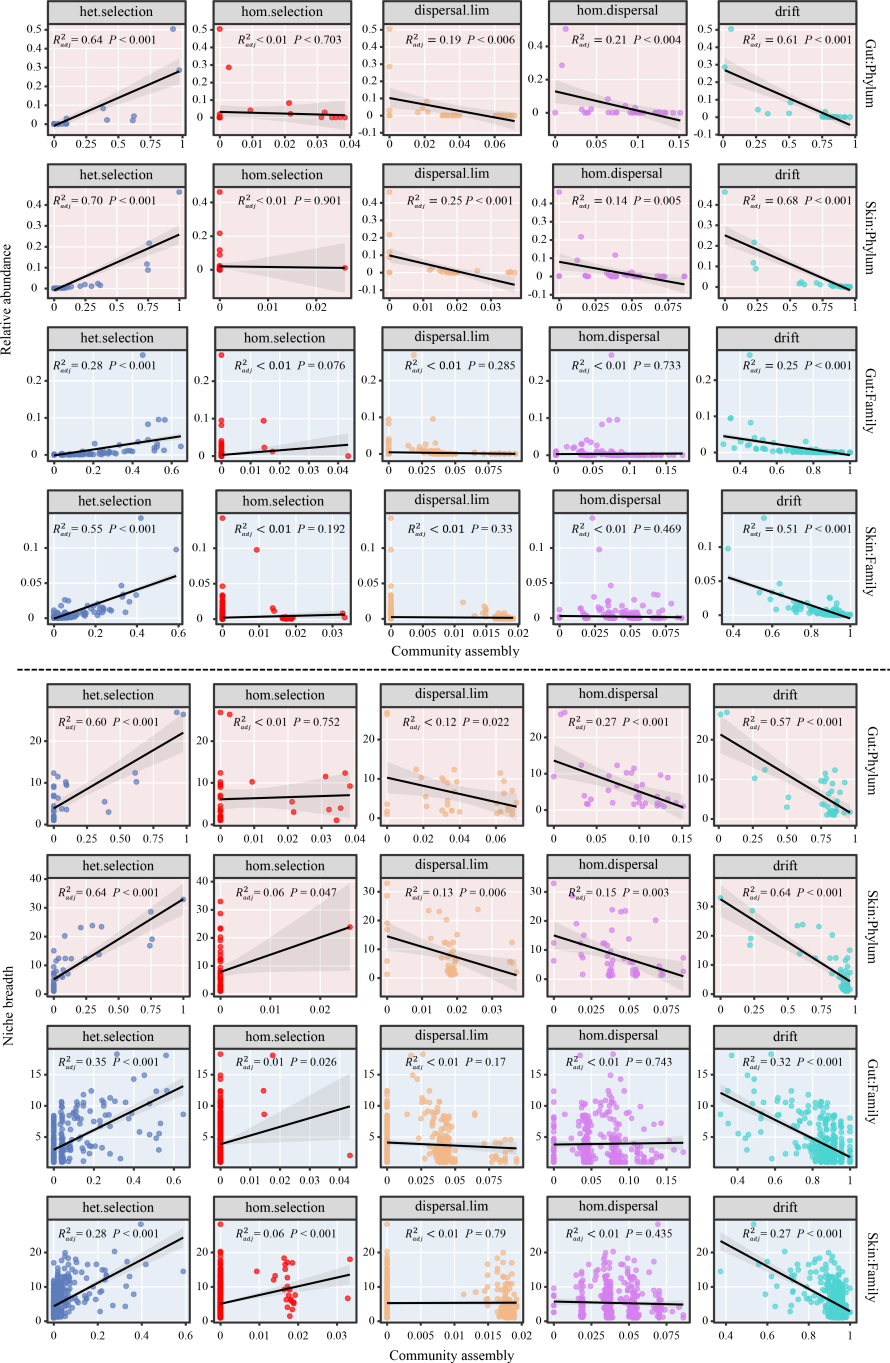
Relationships between the relative abundance (**A**) and niche breadth (**B**) of gut and skin microbes and distinct community assembly processes at the phylum and family levels. The solid black lines denote the fitted ordinary least-squares model, and the gray areas correspond to 95% confidence intervals of the predictor?

Finally, an important pattern was revealed in that both the relative abundance and niche breadth of taxa were markedly positively correlated with the relative contribution of heterogeneous selection, and negatively correlated with drift (all *P* < 0.05, Fig. 5), implying that community assembly processes are strongly engaged in the regulation of symbiotic microbial community structure.

### Environmental variables influenced symbiotic microbial community structure and assembly

**Fig 6:**
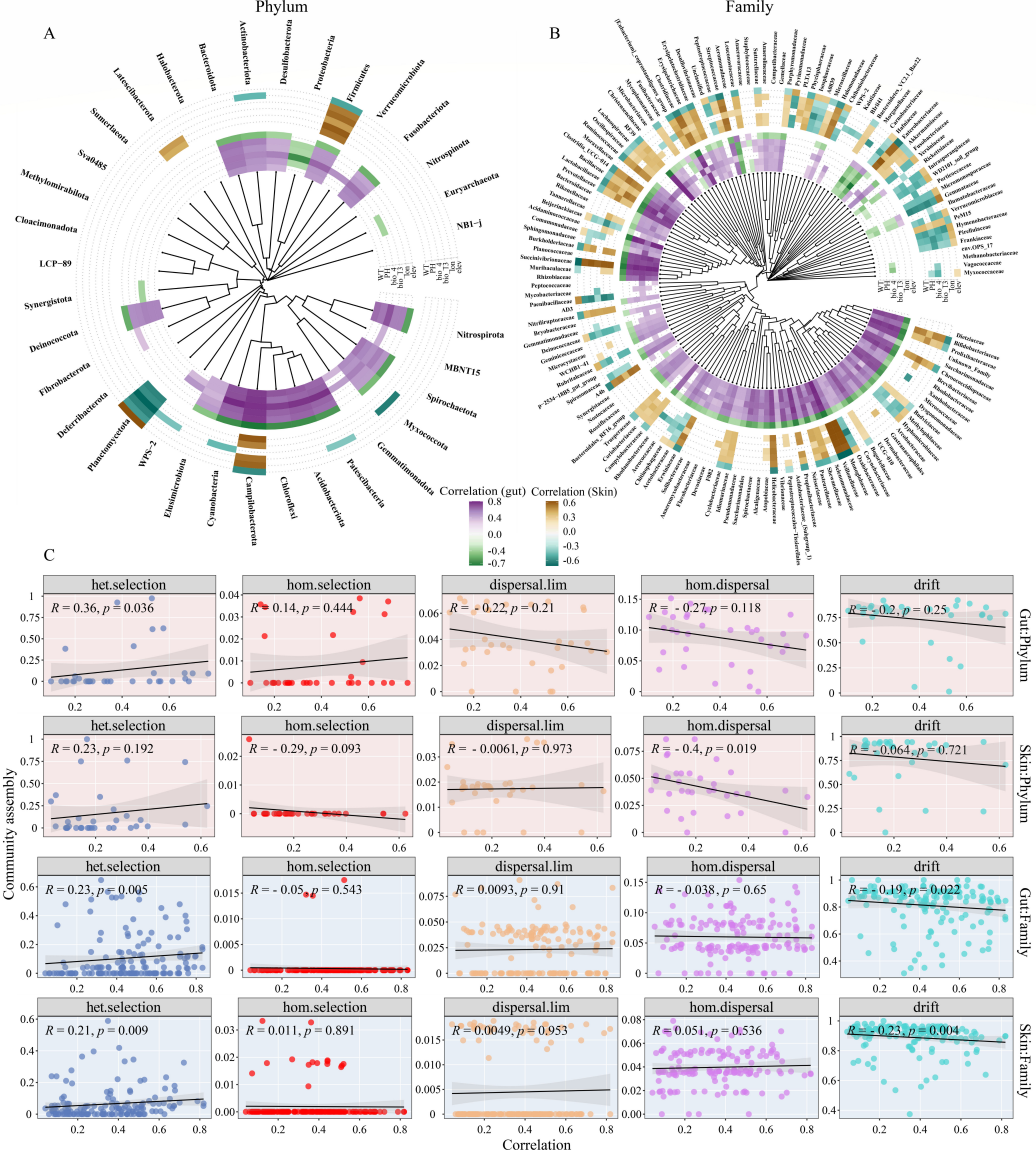
Spearman’s rank correlation of environmental variables with a relative abundance of symbiotic microbes at the phylum (**A**) and family (**B**) levels (only taxa with *P* < 0.05 are shown). The innermost heat map displays the relative abundance of gut microbes in relation to environmental factors, while the outermost heat map represents the skin microbes. (**C**) Spearman’s rank correlation of distinct community processes with the correlations (mean of absolute values of fitness values in all environmental factors) between environmental factors and microbial taxa relative abundance.

We adopted Random Forest (RF) models to reveal the effects of environmental variables on the structure of symbiotic microbial communities and their relative importance (Gut: %Var explained = 60.43, Skin: %Var explained = 37.28; Fig. S5). The results showed that bio_4 was the most important variable for gut microbial richness, while longitude was the most important variable for skin microbial richness. Importantly, we observed that the relative abundance of most phyla and families was significantly correlated with environmental variables (Fig. 6A and B), with most taxa negatively correlated with altitude (elev) and positively correlated with the other five factors (WT, PH, bio_4, bio-13, lon), suggesting that environmental factors strongly affected the structure of the symbiotic microbial community at different altitude gradients. However, the Planctomycetota phylum and the Hafniaceae dominant family were negatively correlated with these five environmental factors and positively correlated with altitude.

More importantly, the community assembly processes markedly manipulate the correlation between environmental factors and the relative abundance of the taxon (Fig. 6C; Fig. S6 to S9). On the whole, we examined that heterogeneous selection was positively correlated with the correlations between environmental factors and taxa relative abundance, and negatively correlated with drift, excluding phylum-level skin microbes (Fig. 6C). However, at the phylum-level skin microbes, we found a significant negative correlation between homogenizing dispersal and their correlations. Concretely, at the phylum level, we detected that heterogeneous selection was positively correlated with the correlations between bio_4 and taxa relative abundance (Fig. S6). At the family level in gut microbes, only a significant positive correlation of bio_4 with heterogeneous selection and a negative correlation with drift was detected (Fig. S8). However, in skin microbes, there was no significant correlation between heterogeneous selection and bio_4, but significant positive correlations with WT, PH, bio_13, and lon and significant negative correlations with elev (Fig. S9). Notably, although the other three community assembly processes were not significantly correlated with the correlations between environmental factors and taxa relative abundance but there were relatively high correlation values. MRM analysis showed (Fig. S10) that for gut microbes, both altitude and habitat type had a significant effect on their symbiotic microbial diversity (α, R^2^ = 0.16, *P* = 0.001; β, R^2^ = 0.21, *P* = 0.001). For skin microbes, only elevation and species were found to have a significant effect on β diversity (R^2^ = 0.37, *P* = 0.001), with altitude as the most important effect.

## DISCUSSION

Individual microorganisms or groups, along with sampled areas, provide valuable tools for diverse applications including assessing microbiological or habitat quality and informing environmental management strategies (35, 36). For the research topic investigated here, microbial indicator taxa and sampling units can shed light on the underpinning ecological mechanisms maintaining microbial community patterns. Our findings revealed that among the five mechanisms examined, heterogeneous selection and drift were the principal determinants of microbial community structure for groups of DTUs, whereas homogeneous and heterogeneous selection are dominant drivers of groups of DSUs (Fig. 2D, 3, and 4).

The indicative DTU and DSU analyses employed in our study diverge from the conventional methods used in previous microbial studies (35, 37). For instance, traditional indicator taxa analysis employed in microbial and macro-organismal community ecology studies followed the rationale that a taxon with a high indicator value should have a high probability of being present in a targeted sample and absent elsewhere (35, 37–39). However, for the indicator taxa analysis developed in this study, we focused on the unique contribution of each taxon in identifying evidence of selection, dispersal, and drift within the entire microbial community. The indicator taxa analysis is specially tailored for Stegen et al.’s analytical framework (3, 9) for distinguishing selection, dispersal, and drift on the microbial community structure. A taxon is a useful indicator if its absence can alter conclusions with respect to the various underlying mechanisms that are exerted on the pair of sites. For example, a taxon serves as an indicator of heterogeneous selection for a particular pair of sites if its presence renders the metric significant, whereas its absence leads to nonsignificant (see decision rule 1).

The taxon removal algorithm has been applied in community ecology and microbial ecology with different purposes, such as assessing food web stability, evaluating ecosystem functioning performance, or determining the metabolic roles of gut microbiomes (40, 41). Our study incorporates the taxon-specific removal algorithm into the randomization tests to identify taxa that can remarkably contribute to the underlying mechanisms shaping the microbial community structure and beta diversity patterns (Fig. 2A). Although the study objectives might be different, the central goal of applying the taxon removal algorithm is to identify keystone microbial taxa associated with community stability, biodiversity maintenance, ecosystem functioning, or environmental issues (40, 42, 43). Moreover, our site removal algorithm has similar functions, such as identifying crucial sites that uphold microbial community structure and microbial ecosystem services. Environmental heterogeneity can remarkably shift the structure of amphibian symbiotic microbial communities (13, 44), which is consistent with our results, but this difference lacks a clearer explanation at the community assembly. Here, our contribution analysis of groups of DSUs and DTUs can provide a deeper explanation of community-level findings (Fig. 2 to 4). For DSUs in different altitude groups (Fig. 2D), we discovered that heterogeneous and homogeneous selection dominated the community assembly of symbiotic microbes, but stochastic processes tended to increase with altitude, which may account for the significant differences in the α- and β-diversity of symbiotic microbes at different altitude gradients (Fig. 2D and E). Also, this may be explained by the fact that amphibians are the taxa most sensitive to environmental change versus other vertebrates (45, 46). Notably, the community assembly processes at mid and high altitudes were in fact relatively less different than those at lower altitudes, which can be attributed to similar environmental stresses experienced by amphibians after reaching a certain altitude, resulting in parallel processes of symbiotic microbial community assembly.

In this research, the composition and diversity of symbionts at different altitudinal gradients show variability, and significant divergence between skin and gut microbes, implying that these symbiotic taxa play different roles in the adaptation of hosts to different altitudinal habitats. Meanwhile, these differences may be mediated by different community assembly processes. Hence, we used a contribution analysis of DTUs to explore the driving community assembly processes behind these differences, particularly for some indicator taxa. Overall, we found that drift was the dominant mechanism driving the community structure in the most symbiotic microbial taxa, while heterogeneous selection governed community assembly in most dominant or indicator taxa (Fig. 3 and 4), as the reason may be because most indicator species are the result of self-selection by their hosts for specific habitats, and because host selection is an important deterministic process (47, 48). Interestingly, we also found that these heterogeneous selection-dominated indicator taxa have relatively broader niche breadth, which may be justified by the fact that the broader the niche breath of a species, it becomes more competitive, more widely distributed, and more abundant (2, 49).

In niche-based theory, environmental filtering (e.g., environmental factors) and biological interactions (e.g., host selection, competition) largely govern the structure of microbial communities (3, 4, 50). This is again evidenced in our results where distinct environmental factors are significantly correlated with the relative abundance of species (Fig. 6A and B) and community assembly processes strongly mediate the interaction between environmental variables and taxon abundance (Fig. 6C; Fig. S6 to S9), suggesting in-depth that habitat heterogeneity across elevational gradients or heterogeneity in host selection plays an important role in shaping different symbiotic microbial communities. In addition, a significant correlation was observed between differences in the structure of the skin and gut microbial communities and differences in its community assembly processes (Fig. S4), showing that variations in community assembly processes can shape different symbiotic microbial communities. Previous studies of community assembly have primarily focused on the grouping level of DSUs (3, 4, 48), and here we observed vital community patterns from the DTU level (Fig. 5): the niche breadth and relative abundance of microbial taxa are significantly negatively correlated with drift, and positively correlated with heterogeneous selection, which might be the result of host selection (50, 51) and the higher niche breadth and ecological fitness of dominant taxa (Fig. 3 and 4). Interestingly, we detected a significant positive correlation between niche breadth and relative abundance of symbiotic microbial taxa, with a markedly higher correlation at the phylum level than at the family level (Fig. S3). In addition, the effect of altitude appeared to be more important than the effect of species and habitat type on symbiotic microbial diversity (Fig. S10), implying that altitude is an important driver of differences in symbiotic biodiversity along the altitudinal gradient and that grouping by altitudinal gradient is more reasonable in this study. In addition, although the effect of species on the beta diversity of symbiotic microbial was weaker versus altitude, it should be noted that this suggests that species also has an effect on symbiotic microbial communities, which can be further explored in future research. In contrast to previous studies (2, 9, 48), we took a deep dive into different community assembly processes that significantly control the structure of symbiotic microbial communities and regulate their interactions with environmental factors at the level of DSUs and DTUs. We also revealed that the extent of this regulation varies depending on the host habitat. Also, this is evidenced that different community assembly processes may be able to regulate the structure of the symbiotic microbial community to facilitate better adaptation of the host to different habitat gradients. More importantly, our modeling framework has two key advantages over earlier methods (3, 4, 9) for community assembly analysis, one being the quantitative relative contribution of DTUs and the other being the quantitative relative contribution of individual DSUs for many subsequent multivariate statistical analyses.

Our framework can be immediately applied to each individual or specific group of DTUs/DSUs, rendering our approach general, flexible, and appealing for conducting hypothesis testing. It is interesting to compare how the functional role of a specific group of DTUs (e.g., from a specific phylum or class) is related to its uneven contribution to selection, dispersal, and drift. Moreover, it is perhaps equally valuable to compare different groups of DSUs in terms of their relative contributions to different mechanisms. It has been shown that functional redundancy is important to maintain microbial community stability (40, 52). To this end, for future application studies, it is both necessary and valuable to evaluate the joint contribution of a group of microbial taxa in different underlying community assembly rules.

## Data Availability

The raw sequence data and relevant files have been deposited in the NCBI database under accession numbers PRJNA889361 and PRJNA1202351.

## SUPPLEMENTAL MATERIAL

Supplemental figures and tables spectrum.04192-23-s0001.docx Fig. S1 to S10; Tables S1 to S4.

Model R code spectrum.04192-23-s0002.docx The detailed calculation R codes.

ASM does not own the copyrights to Supplemental Material that may be linked to, or accessed through, an article. The authors have granted ASM a non-exclusive, world-wide license to publish the Supplemental Material files. Please contact the corresponding author directly for reuse.
