# Supplementary material for: A novel framework unveiling the importance of heterogeneous selection and drift on the community structure of symbiotic microbial indicator taxa across altitudinal gradients in amphibians
Source: Microbiol Spectr. 2025 Jan 8;13(2):e04192-23. doi: 10.1128/spectrum.04192-23 (PMC11792505; doi:10.1128/spectrum.04192-23)
Supplement: Supplemental figures and tables — Fig. S1 to S10; Tables S1 to S4. [file spectrum.04192-23-s0001.docx]

Supporting Information

A novel framework unveiling the importance of heterogeneous selection and drift on the community structure of symbiotic microbial indicator taxa across altitudinal gradients in amphibians

**Additional figures and tables**

**Fig. S1. Schematic and definitions of microbial community assembly processes.**

**
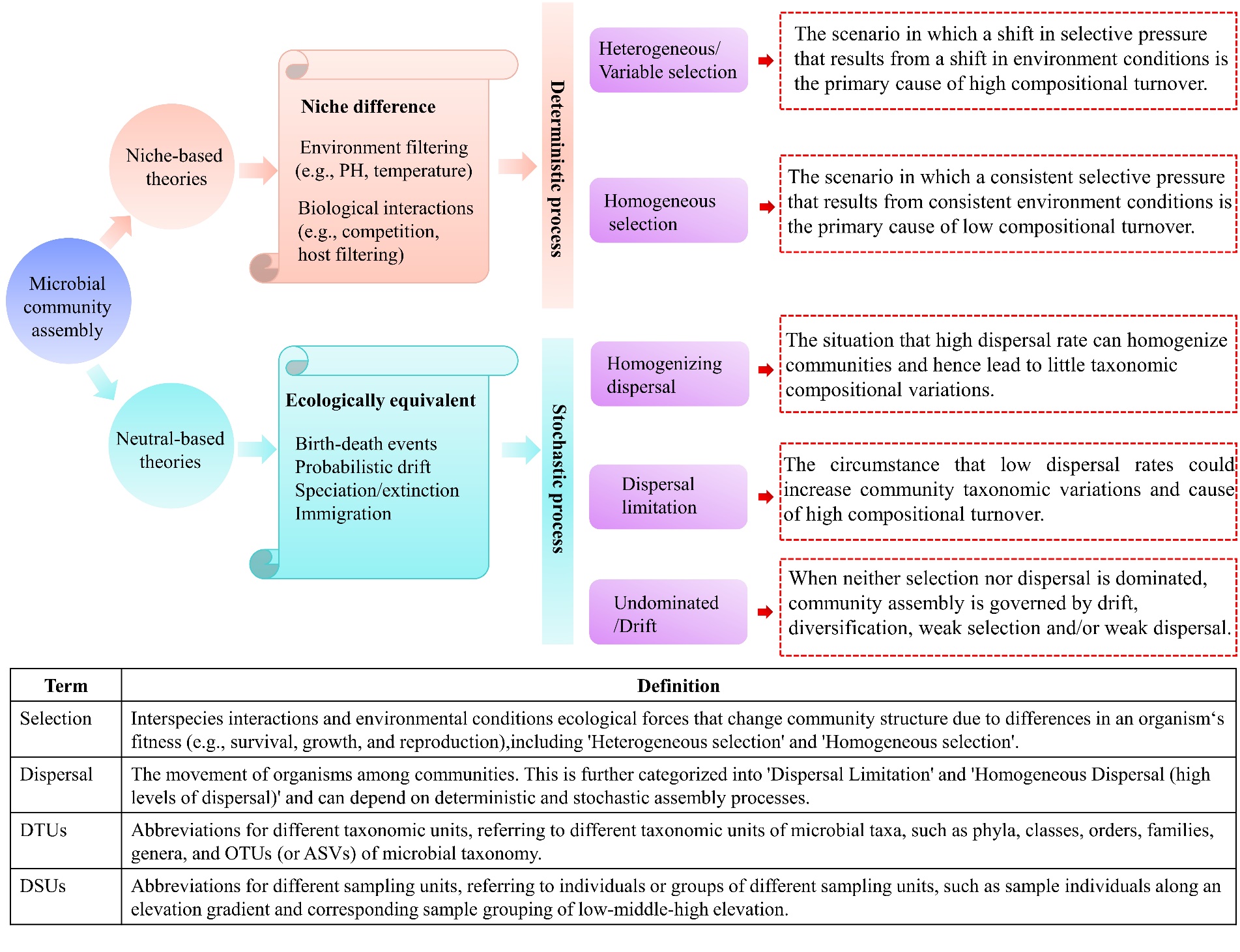
**

Fig. S2. Heat map of species distribution for the top 30 phyla (A) and families (B) in relative abundance along an altitudinal gradient. L_G, M_G, H_G represent gut microbes at low-, mid- and high-altitudes, while L_SK, M_SK, H_SK represent skin microbes at low-, mid- and high-altitudes, respectively.


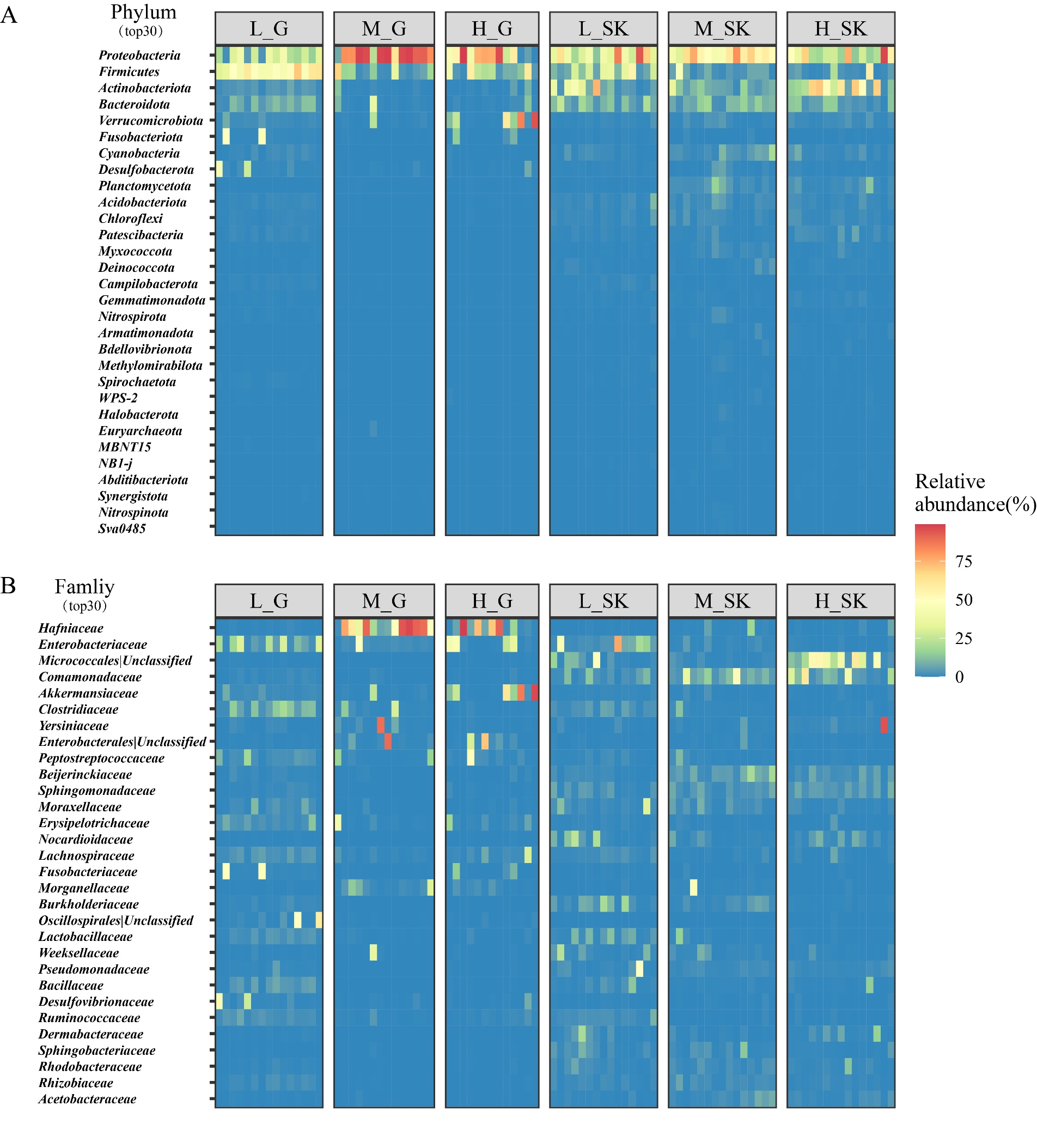


Fig. S3. Relationships of niche breadth with relative abundance in gut and skin microbes at phylum and family level. The solid lines represent the fitted ordinary least-squares model, and the grey areas correspond to 95% confidence intervals of the predictor.


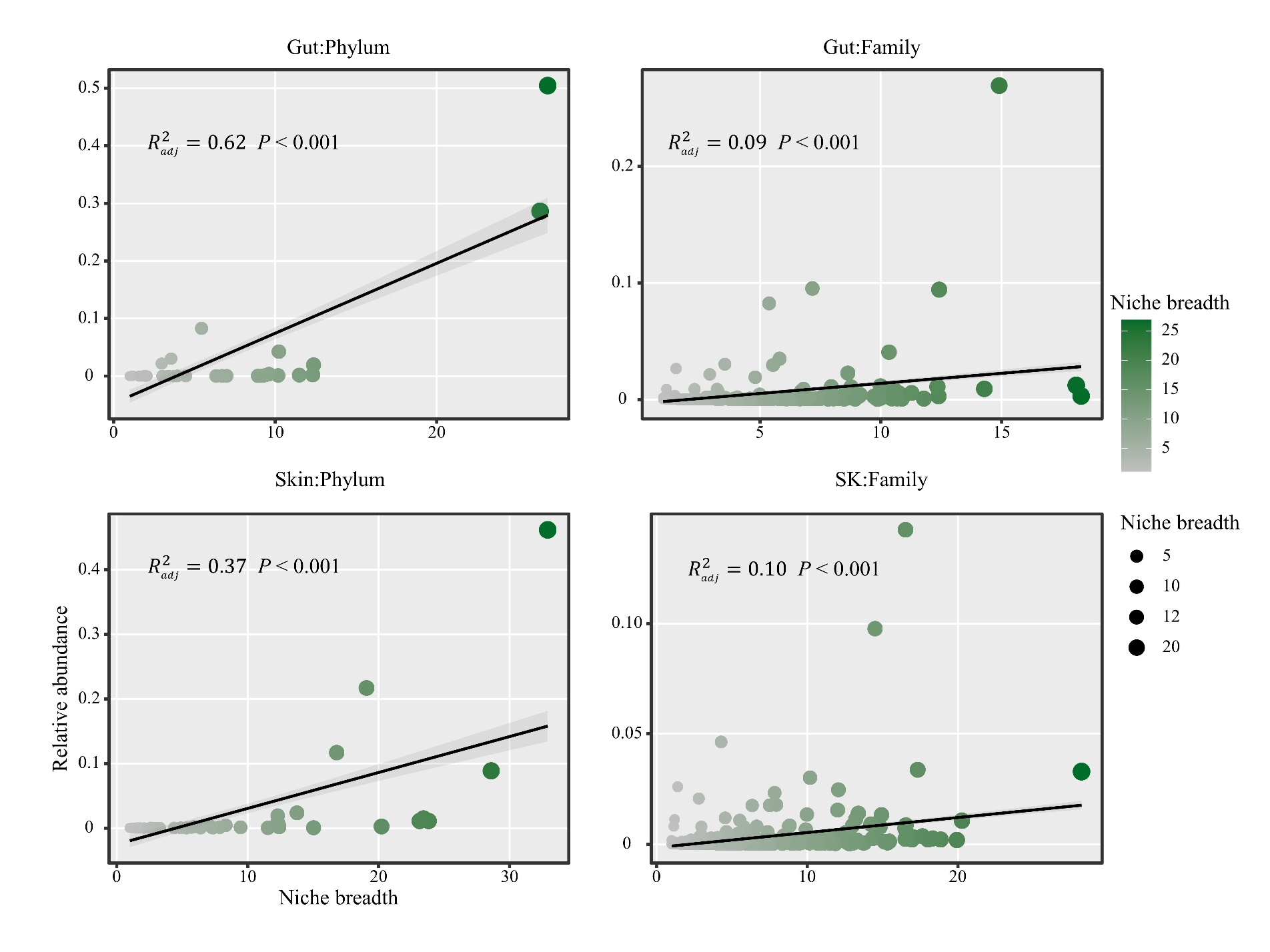


Fig S4. Relationships of difference in distinct community processes with variations in the different properties (relative abundance and niche breadth) in gut and skin microbes at phylum and family level. (A) Difference in niche breadth at the phylum level. (B) Difference in relative abundance at the phylum level. (C). Difference in niche breadth at the family level. (D) Difference in relative abundance at the family level. The solid lines represent the fitted ordinary least-squares model, and the grey areas correspond to 95% confidence intervals.


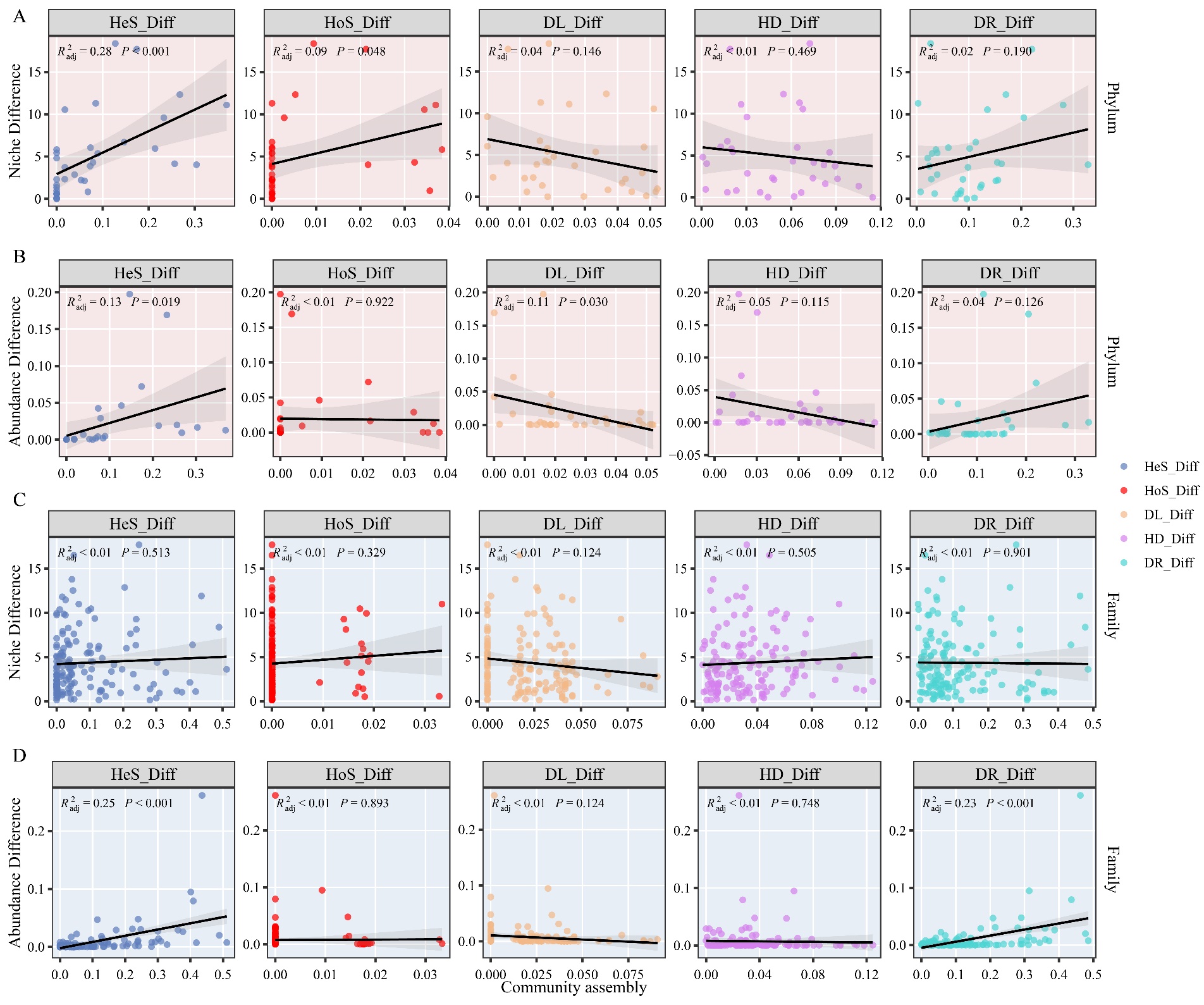


Fig S5. Potential environmental drivers for the richness difference of amphibian gut (A) and skin (B) microbes along altitudinal gradient. Percentage increases in the MSE (%IncMSE) of variables were used to estimate the importance of these predictors, and higher %IncMSE values imply more important predictors. Differences are denoted as follows: ∗p < 0.05; ∗∗p< 0.01; ∗∗∗p < 0.001; ns represent non-significant. MSE = mean squared error. %Var explained: overall explanation of variance.


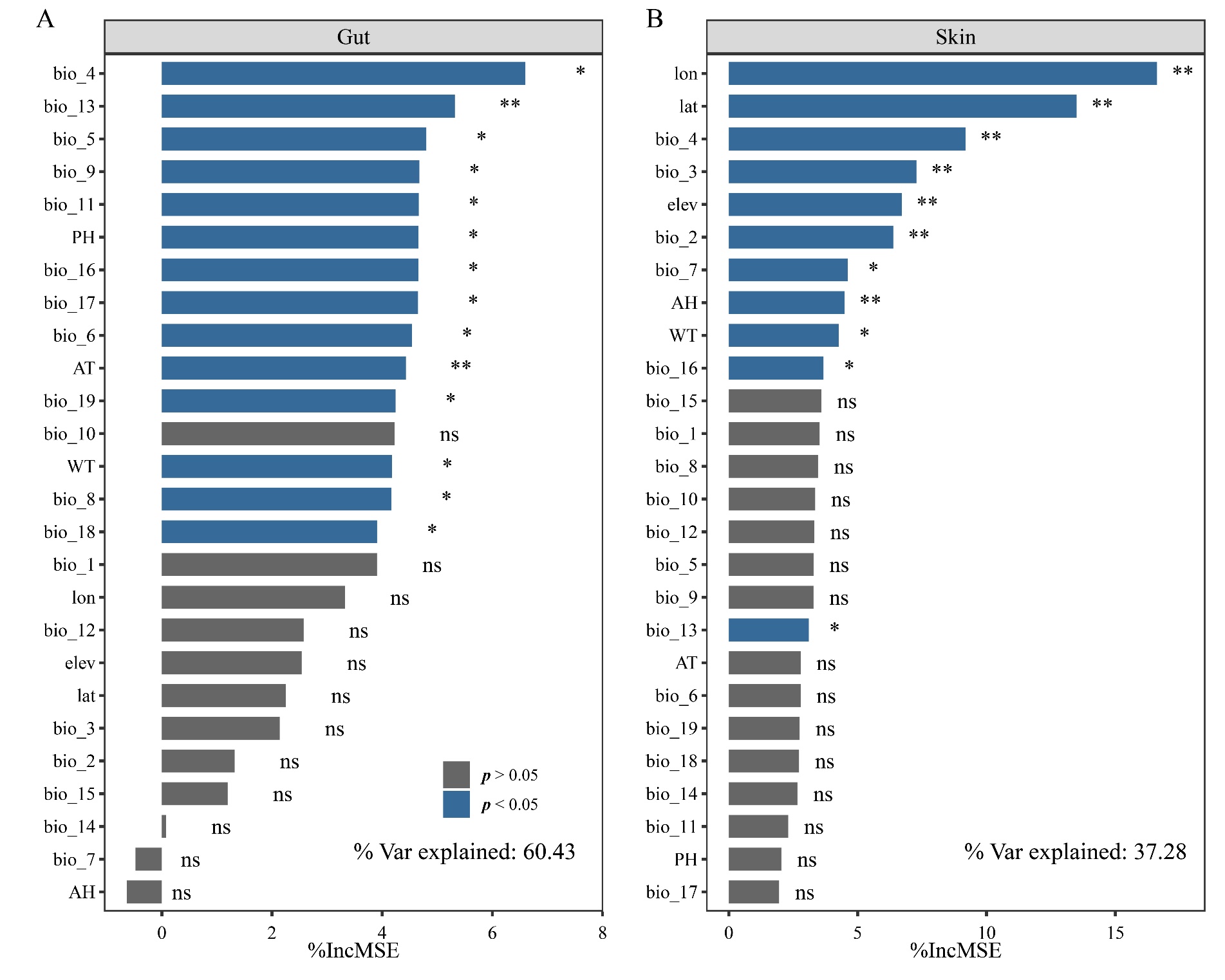


Fig S6. Spearman’s rank correlation of distinct community processes with the correlations (absolute values of fitness values in each environmental factor) between environmental factors and taxa relative abundance of gut microbes at the phylum level.


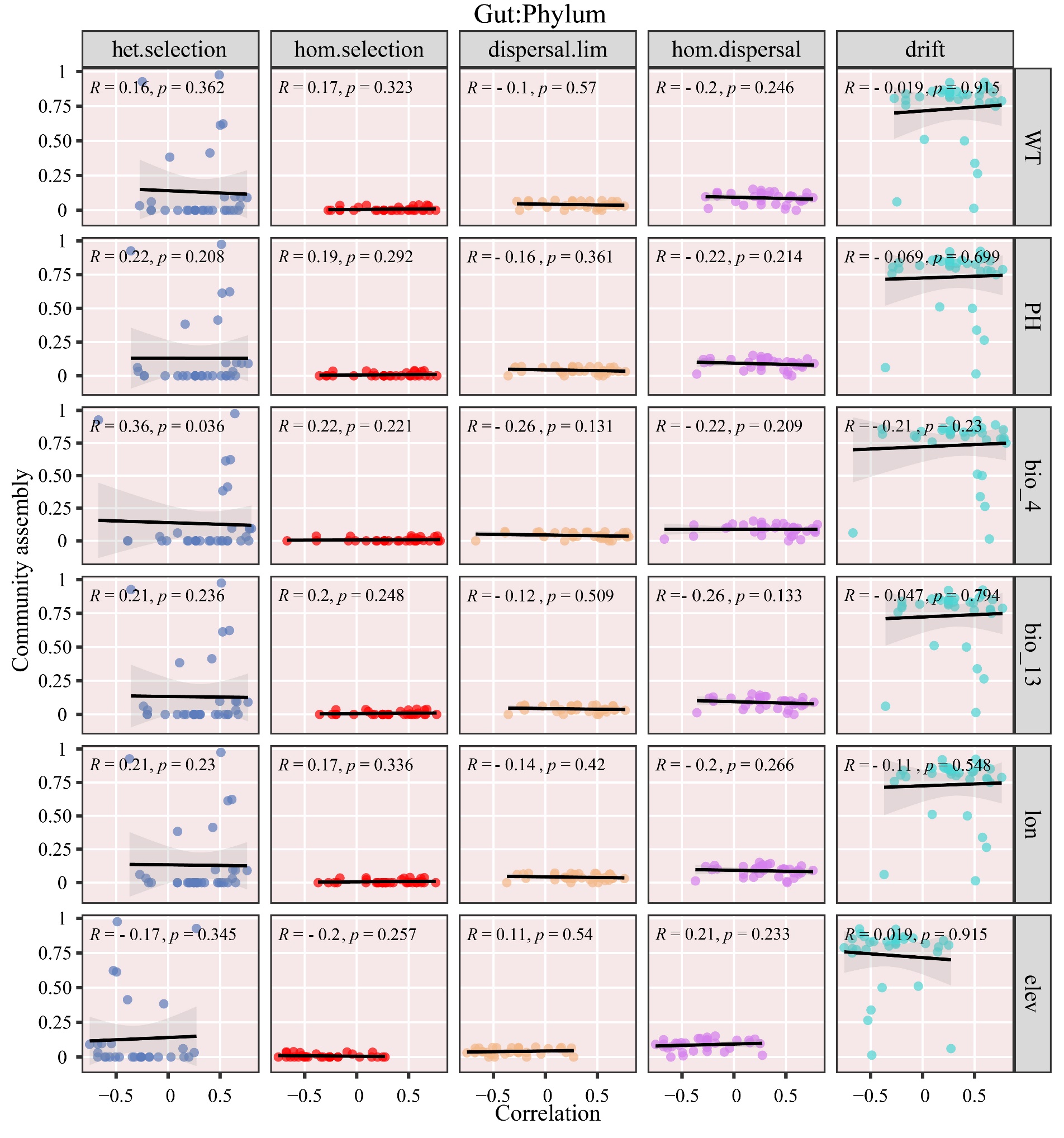


Fig S7. Spearman’s rank correlation of distinct community processes with the correlations (absolute values of fitness values in each environmental factor) between environmental factors and taxa relative abundance of skin microbes at the phylum level.


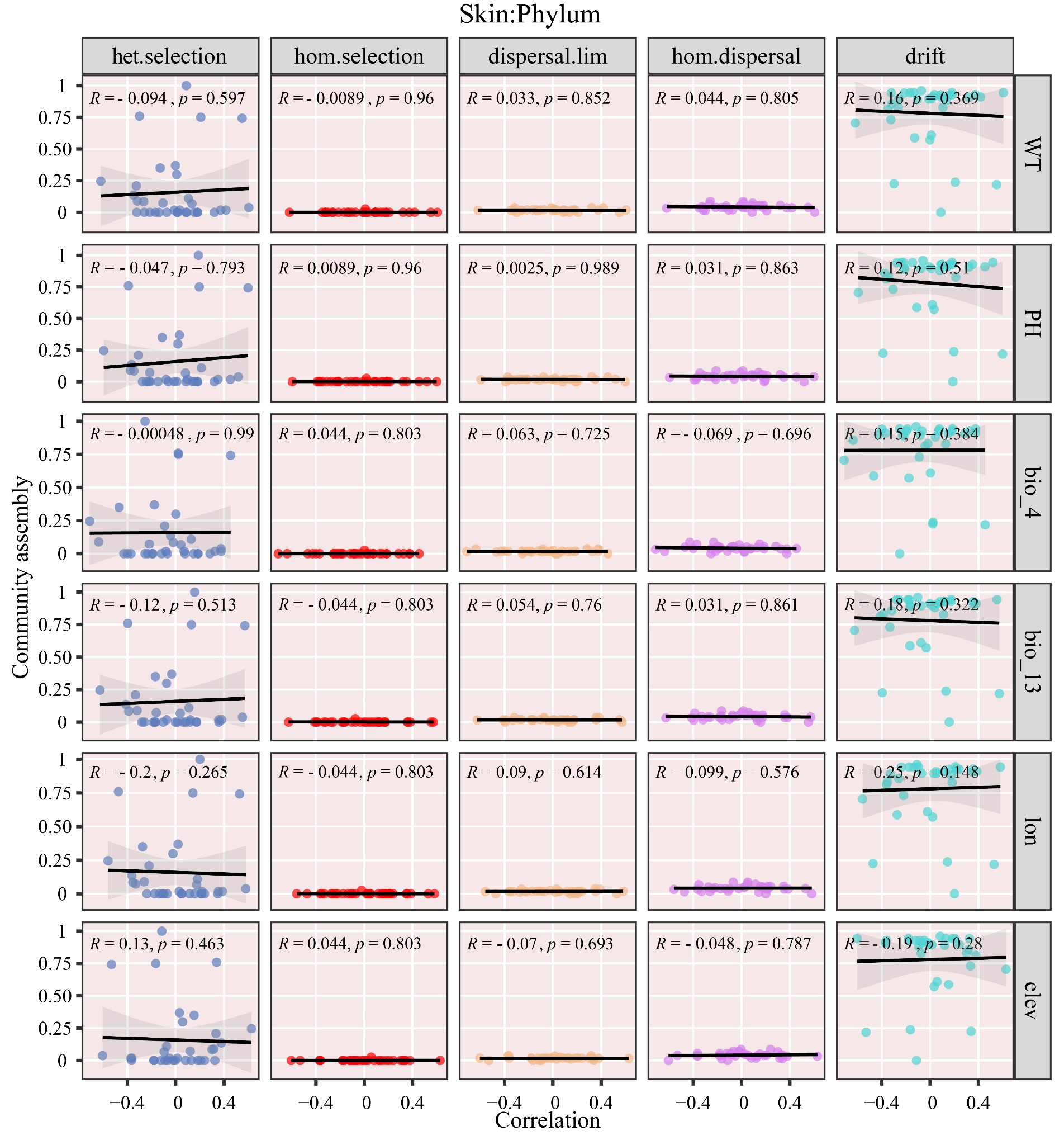


Fig S8. Spearman’s rank correlation of distinct community processes with the correlations (absolute values of fitness values in each environmental factor) between environmental factors and taxa relative abundance of gut microbes at the family level.


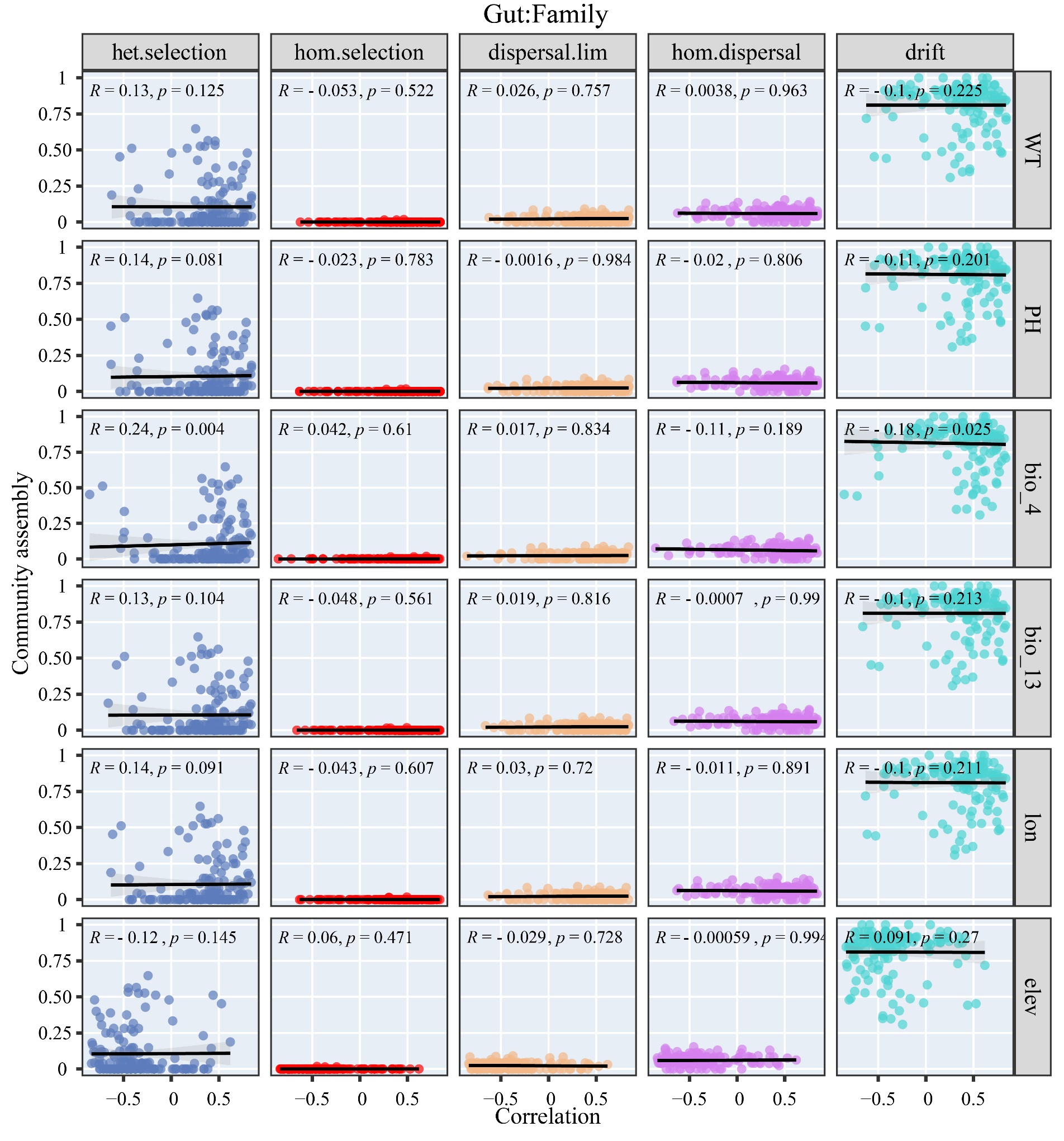


Fig S9. Spearman’s rank correlation of distinct community processes with the correlations (absolute values of fitness values in each environmental factor) between environmental factors and taxa relative abundance of skin microbes at the family level.


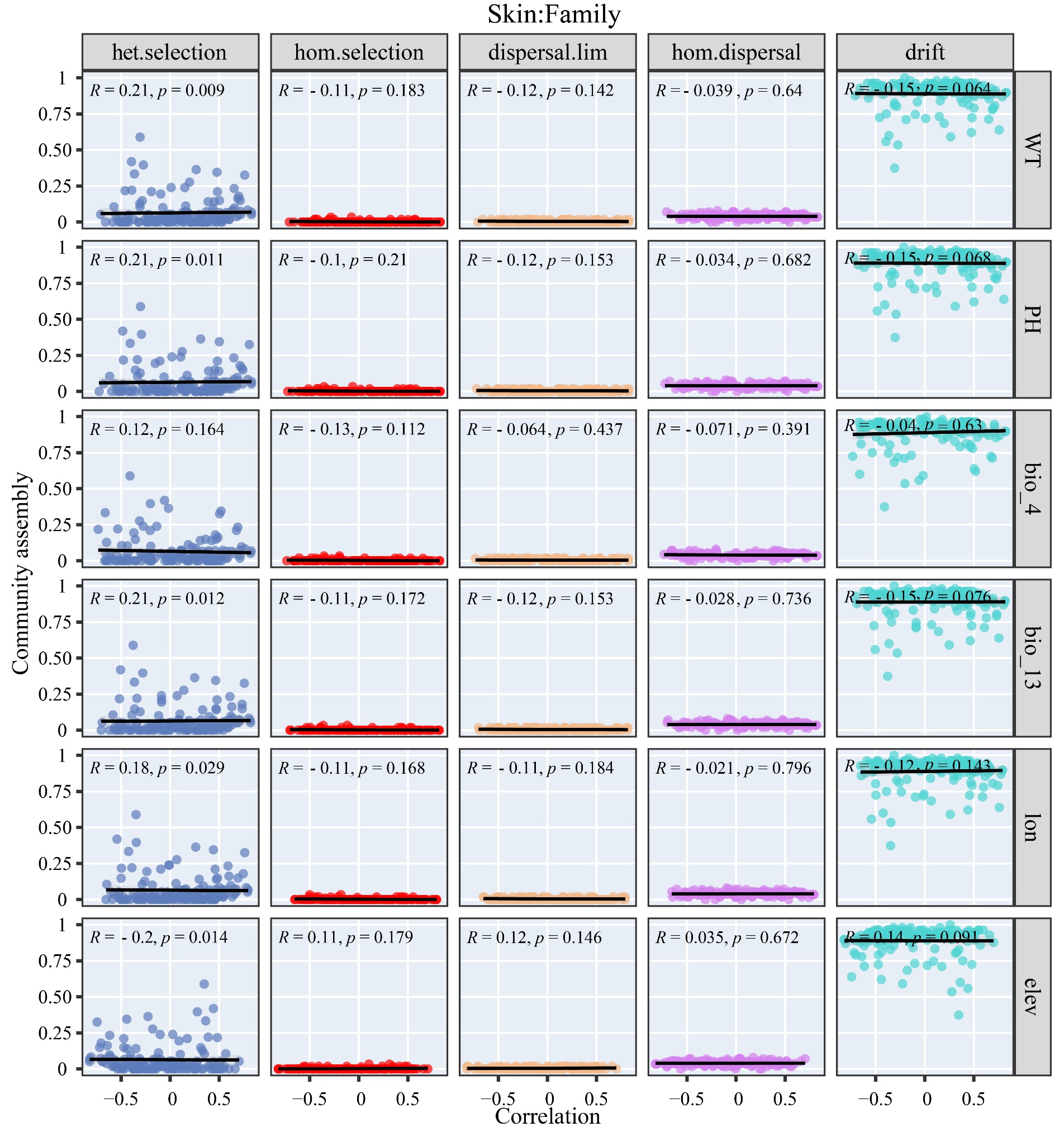


Fig S10. Altitude, species, and habitat type influence the extent of gut and skin microbial diversity. The bar plots show the absolute values of the partial regression coefficients for the different variables in the MRM analysis, with the annotations above them indicating the significance of the variables. Differences are denoted as follows: ∗*p* < 0.05; ∗∗*p*< 0.01; ∗∗∗*p* < 0.001; ns represent not significant. R^2^ denotes the global effect value of the MRM model.


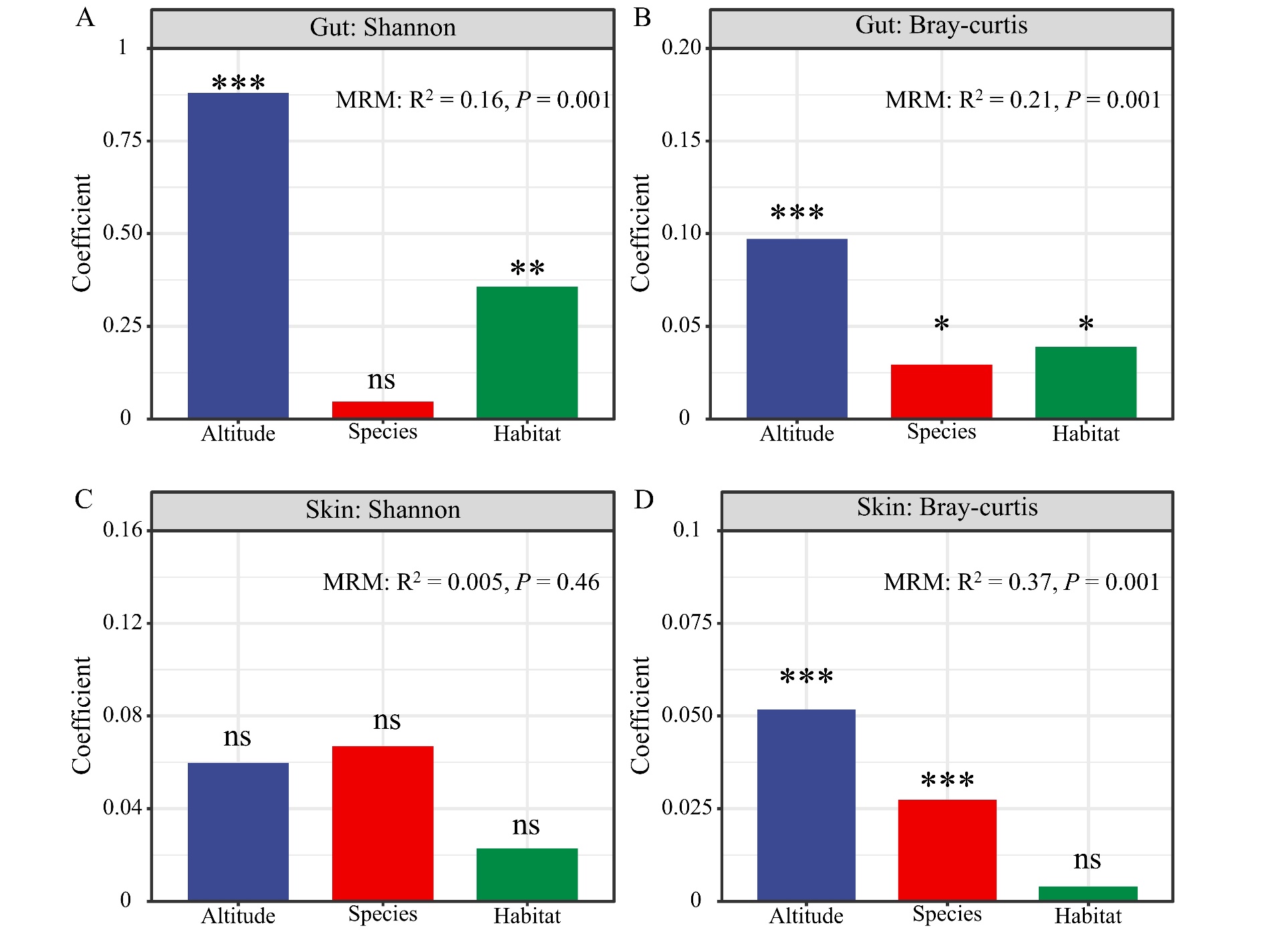


Table S1. Sampling locations of the amphibian symbiotic microbes. N_skin_ refers to the number of skin microbial samples, while N_gut_ denotes the number of gut microbial samples.

| Group | N_gut_ | N_sk_ | Longtitude | Latitude | Altitude (m) | Habitat types | Species |
| --- | --- | --- | --- | --- | --- | --- | --- |
| L | 15 | 15 | 104.05258 | 30.57301 | 439 | Semi-aquatic | *Pelophylax nigromaculatus* |
| M | 14 | 15 | 102.48832 | 29.05532 | 2141 | Terrestrial | *Rana kukunoris* |
|  |  |  |  |  |  | Semi-aquatic | *Amolops mantzorum* |
|  |  |  |  |  |  |  | *Amolops loloensis* |
| H | 13 | 15 | 100.32216 | 29.59666 | 3880 | Terrestrial | *Scutiger boulengeri Bufo gargarizans* |
|  |  |  | 100.14186 | 30.05564 | 3989 |  |  |
|  |  |  | 99.85156 | 30.26418 | 4411 |  |  |

Abbreviations: N_gut_: Number of gut samples; N_sk_, Number of gut samples.

Table S2. List of abbreviations for environmental variables used for analysis.

| Abbreviation | Complete name |
| --- | --- |
| bio_1 | Annual Mean Temperature |
| bio_2 | Mean Diurnal Range (Mean of monthly (max temp - min temp)) |
| bio_3 | Isothermality (bio_2/bio_7) (×100) |
| bio_4 | Temperature Seasonality (standard deviation ×100) |
| bio_5 | Max Temperature of Warmest Month |
| bio_6 | Min Temperature of Coldest Month |
| bio_7 | Temperature Annual Range (bio_5–bio_6) |
| bio_8 | Mean Temperature of Wettest Quarter |
| bio_9 | Mean Temperature of Driest Quarter |
| bio_10 | Mean Temperature of Warmest Quarter |
| bio_11 | Mean Temperature of Coldest Quarter |
| bio_12 | Annual Precipitation |
| bio_13 | Precipitation of Wettest Month |
| bio_14 | Precipitation of Driest Month |
| bio_15 | Precipitation Seasonality (Coefficient of Variation) |
| bio_16 | Precipitation of Wettest Quarter |
| bio_17 | Precipitation of Driest Quarter |
| bio_18 | Precipitation of Warmest Quarter |
| bio_19 | Precipitation of Coldest Quarter |
| lat | Latitude |
| lon | Longitude |
| elev | Elevation |
| AH | Air humidity |
| AT | Air temperature |

Table S3. Alpha diversity (richness, Shannon index and phylogenetic diversity: PD) of symbiotic microbes at different altitudinal gradients. L_G, M_G, H_G represent gut microbes at low-, mid- and high-altitudes, while L_SK, M_SK, H_SK represent skin microbes at low-, mid- and high-altitudes, respectively. The Sd denotes standard deviation.

| Group | Index | Mean | Sd |
| --- | --- | --- | --- |
| L_G | Richness | 340.13 | 89.36 |
| M_G | Richness | 122.36 | 27.99 |
| H_G | Richness | 124.54 | 51.16 |
| L_SK | Richness | 370.27 | 134.68 |
| M_SK | Richness | 654.80 | 266.89 |
| H_SK | Richness | 573.53 | 168.38 |
| L_G | Shannon | 4.14 | 1.16 |
| M_G | Shannon | 1.75 | 0.58 |
| H_G | Shannon | 1.85 | 0.90 |
| L_SK | Shannon | 4.15 | 0.98 |
| M_SK | Shannon | 5.11 | 0.78 |
| H_SK | Shannon | 3.88 | 1.29 |
| L_G | PD | 39.41 | 5.65 |
| M_G | PD | 12.99 | 3.05 |
| H_G | PD | 15.51 | 4.98 |
| L_SK | PD | 42.47 | 11.70 |
| M_SK | PD | 50.15 | 13.83 |
| H_SK | PD | 46.74 | 10.26 |

Table S4. Beta diversity (Bray–Curtis distance) of symbiotic microbes at different altitudinal gradients. L_G, M_G, H_G represent gut microbes at low-, mid- and high-altitudes, while L_SK, M_SK, H_SK represent skin microbes at low-, mid- and high-altitudes, respectively. The Sd denotes standard deviation.

| Group | Mean | Sd |
| --- | --- | --- |
| L_G | 0.82 | 0.15 |
| M_G | 0.69 | 0.24 |
| H_G | 0.85 | 0.23 |
| L_SK | 0.85 | 0.11 |
| M_SK | 0.90 | 0.07 |
| H_SK | 0.82 | 0.18 |
