# Supplementary material for: A novel framework unveiling the importance of heterogeneous selection and drift on the community structure of symbiotic microbial indicator taxa across altitudinal gradients in amphibians
Source: Microbiol Spectr. 2025 Jan 8;13(2):e04192-23. doi: 10.1128/spectrum.04192-23 (PMC11792505; doi:10.1128/spectrum.04192-23)
Supplement: Model R code — The detailed calculation R codes. [file spectrum.04192-23-s0002.docx]

**Title: A novel framework unveiling the importance of heterogeneous selection and drift on the community structure of symbiotic microbial indicator taxa across altitudinal gradients in amphibians**

Jin Zhou^1,2,3^, Zhidong Liu^1^, Sishuo Wang^4^, Jing Li^2^, Lin Zhang^1*^, Ziyan Liao^1*^

^1^ Chengdu Institute of Biology, Chinese Academy of Sciences, Chengdu, 610041, China

^2^Key Laboratory of Bio-Resources and Eco-Environment of Ministry of Education, College of Life Sciences, Sichuan University, Chengdu, Sichuan 610065, China

^3^University of Chinese Academy of Sciences, Beijing 100049, China

^4^Department of Microbiology, The Chinese University of Hong Kong, Shatin, Hong Kong SAR, China

*corresponding email: [liaozy@cib.ac.cn](mailto:liaozy@cib.ac.cn); [zhanglin@cib.ac.cn](mailto:zhanglin@cib.ac.cn)

Jin Zhou and Zhidong Liu contributed equally to this article.

Here, we propose a comprehensive framework for identifying distinct sampling units (DSUs) /distinct taxonomic units (DTUs) that remarkably contribute to the various mechanisms (community assembly processes) sustaining microbial community structure. The detailed calculation R codes are listed below:

#===============================================================================

# Load Packages

#------------------

library(iCAMP)

library(data.table)

library(picante)

library(vegan)

library(ape)

library(eoffice)

library(foreach) #Parellel run

library(doParallel)

library(plyr)

library(fs)

library(dplyr)

library(wrswoR)

#=====================================================================

# 1.pair.ID() function

#=====================================================================

#pairs of sites ID

pair.ID<-function(sites)

{

n=length(sites)

xy=expand.grid(1:n,1:n)

ids=which(xy[,1]<=xy[,2])

xy=xy[-ids,]

xy1=cbind(sites[xy[,1]],sites[xy[,2]])

#######

return(xy1)

}#

#******************************END*******************************************

#=====================================================================

# 2.dat2group() function

#=====================================================================

#make group list from a dataframe

#the first column is the name of OUT, the second column is the group ID

dat2group<-function(dat)

{

g=unique(dat[,2])

l=list()

for(i in 1:length(g))

{

l[[i]]=dat[which(dat[,2]==g[i]),1]

}#i

return(l)

}#

#******************************END**********************************************

#=====================================================================

# 3.raup_crick() function

#=====================================================================

alpha_table_111 <- function(dataA = bbbb,dataB = aaaa,alpha_levels=alpha_levels,gamma=gamma,occur=occur,rtime=rtime)

{

bb <- as.data.frame(dataA)

names(bb) <- c("a1","a2")

a1 = bb$a1

a2 = bb$a2

aaaa <- dataB

col_count = which(aaaa$a1 == a1 & aaaa$a2 == a2)

#===============================================

##build a null distribution of the number of shared species for a pair of alpha values:

null_shared_spp<-NULL

for(i in 1:rtime){

##two empty null communities of size gamma:

com1<-rep(0,gamma)

com2<-rep(0,gamma)

##add alpha1 number of species to com1, weighting by species occurrence frequencies:

#com1[sample(1:gamma, alpha_levels[a1], replace=FALSE, prob=occur)]<-1

com1[sample_int_rank(gamma, alpha_levels[a1], prob=occur)]<-1

#occupancy=alpha_levelss[a1]

##same for com2:

#com2[sample(1:gamma, alpha_levels[a2], replace=FALSE, prob=occur)]<-1

com2[sample_int_rank(gamma, alpha_levels[a2], prob=occur)]<-1

##how many species are shared in common?

null_shared_spp[i]<-sum((com1+com2)>1)

}

#~~~~~~~~~~~~~~~~~~~~~~~~~~

null_array <- list()

##store null distribution, record values for alpha 1 and 2 in the alpha_table to help find the correct null distribution later:

null_array[[col_count]]<-null_shared_spp

#null_array store null models for each alpha type for a pair of sites

#~~~~~~~~~~~~~~~~~~~~~~~~~~

alpha_table<-data.frame(c(NA), c(NA))

names(alpha_table)<-c("smaller_alpha", "bigger_alpha")

alpha_table[col_count, which(names(alpha_table)=="smaller_alpha")]<-alpha_levels[a1]

alpha_table[col_count, which(names(alpha_table)=="bigger_alpha")]<-alpha_levels[a2]

#place the the alpha_levels to the alpha_table

return(list(alpha_table,null_array))

}

#~~~~~~~~~~~~~~~~~~~~~~~~~~~~~~~~~~~

# (2).raup_crick function

#~~~~~~~~~~~~~~~~~~~~~~~~~~~~~~~~~~~~

# spXsite <- ssmat;rtime=999;plot_names_in_col1=FALSE;classic_metric=FALSE;split_ties=TRUE;set_all_species_equal=FALSE;as.distance.matrix=TRUE;report_similarity=FALSE

raup_crick=function(spXsite,

rtime=999,

#threads=1,

plot_names_in_col1=FALSE,

classic_metric=FALSE,

split_ties=TRUE,

set_all_species_equal=FALSE,

as.distance.matrix=TRUE,

report_similarity=FALSE){#

# library(foreach)

# library(doParallel)

reps=rtime

##this section moves plot names in column 1 (if specified as being present) into the row names of the matrix and drops the column of names

if(plot_names_in_col1==TRUE){

row.names(spXsite)<-spXsite[,1]

spXsite<-spXsite[,-1]

}

## count number of sites and total species richness across all plots (gamma)

n_sites<-nrow(spXsite)

gamma<-ncol(spXsite)

##make the spXsite matrix into a pres/abs. (overwrites initial spXsite matrix):

ceiling(spXsite/max(spXsite))->spXsite

class(spXsite)

##create an occurrence vector- used to give more weight to widely distributed species in the null model:

occur<-apply(spXsite, MARGIN=2, FUN=sum)

#colSums

##NOT recommended- this is a non-trivial change to the metric:

##sets all species to occur with equal frequency in the null model

##e.g.- discards any occupancy frequency information

if(set_all_species_equal){

occur<-rep(1,gamma)

}

## determine how many unique species richness values are in the dataset

##this is used to limit the number of null communities that have to be calculated

alpha_levels<-sort(unique(apply(spXsite, MARGIN=1, FUN=sum)))

#sort alpha_levels from low to high

##make_null:

##alpha_table is used as a lookup to help identify

#which null distribution to use for the tests later.

#It contains one row for each combination of alpha richness levels.

# alpha_table<-data.frame(c(NA), c(NA))

# names(alpha_table)<-c("smaller_alpha", "bigger_alpha")

#

# ##null_array will hold the actual null distribution values. Each element of the array corresponds to a null distribution for each combination of alpha values. The alpha_table is used to point to the correct null distribution- the row numbers of alpha_table correspond to the [[x]] indices of the null_array. Later the function will find the row of alpha_table with the right combination of alpha values. That row number is used to identify the element of null_array that contains the correct null distribution for that combination of alpha levels.

# null_array<-list()

#

##make_null:

##alpha_table is used as a lookup to help identify

#which null distribution to use for the tests later.

#It contains one row for each combination of alpha richness levels.

#-----------------------------------------------------------------

#Make the combined a1 and a2 datasets:分解 a1和a2

aaaa <- data.frame(a1 = NULL, a2 = NULL)

for(a1 in 1:length(alpha_levels)){

for(a2 in a1:length(alpha_levels)){

aaaa <- rbind(aaaa, data.frame(a1 = a1, a2 = a2))

}

}

bbbb <-split(aaaa,1:length(aaaa$a1))#convert the list by row

#--------------------------------------------------------------------------

start2 <- Sys.time()

#The lapply function is used to calculate the parallel function of foreach

data0 <- lapply(bbbb, alpha_table_111,dataB = aaaa,alpha_levels=alpha_levels,gamma=gamma,occur=occur,rtime=rtime)

# data0

Sys.time()-start2

#--------------------------------------------------------------------------

# # length(bbbb)#the numbers of subsets of data0

# #extract the alpha_table from data0

# alpha_table <- foreach(i=1:length(bbbb),.combine = rbind)%do%{

# a <- as.data.frame(data0[[i]][1])[i,]

# return(a)

# }

#

# alpha_table

#----------------------

alpha_table<-data.frame()

for (i in 1: length(bbbb)){

a <- as.data.frame(data0[[i]][1])[i,]

alpha_table = rbind(alpha_table, a)

}

#----------------------------------

# #extract the null_array from data0

# null_array<-list()

# b <- list()

# head(data0)

# null_array <- foreach(i=1:length(bbbb))%do%{

# b[[i]] <- unlist(data0[[i]][2][[1]][i])

# #return(null_array)

# }

# # null_array

# #-----------------------------

null_array<-list()

b <- list()

for (i in 1:length(bbbb)) {

#i=1

b[[i]] <- unlist(data0[[i]][2][[1]][i])

}

null_array <- b

#----------------------------------------------------------

##create a new column with both alpha levels to match on:

alpha_table$matching<-paste(alpha_table[,1], alpha_table[,2], sep="_")

#####################

##do the test:

##build a site by site matrix for the results, with the names of the sites in the row and col names:

results<-matrix(data=NA, nrow=n_sites, ncol=n_sites, dimnames=list(row.names(spXsite), row.names(spXsite)))

##for each pair of sites (duplicates effort now to make a full matrix instead of a half one- but this part should be minimal time as compared to the null model building)

for(i in 1:n_sites){

for(j in 1:n_sites){

##how many species are shared between the two sites:

n_shared_obs<-sum((spXsite[i,]+spXsite[j,])>1)

## what was the observed richness of each site?

obs_a1<-sum(spXsite[i,])

obs_a2<-sum(spXsite[j,])

##place these alphas into an object to match against alpha_table (sort so smaller alpha is first)

obs_a_pair<-sort(c(obs_a1, obs_a2))

##match against the alpha table- row index identifies which element of the null array

#contains the correct null distribution for the observed combination of alpha values:

null_index<-which(alpha_table$matching==paste(obs_a_pair[1], obs_a_pair[2], sep="_"))

##how many null observations is the observed value tied with?

num_exact_matching_in_null<-sum(null_array[[null_index]]==n_shared_obs)

##how many null values are bigger than the observed value?

num_greater_in_null<-sum(null_array[[null_index]]>n_shared_obs)

rc<-(num_greater_in_null)/reps

if(split_ties){

rc<-((num_greater_in_null+(num_exact_matching_in_null)/2)/reps)

}

if(!classic_metric){

##our modification of raup crick standardizes the metric to range from -1 to 1 instead of 0 to 1

rc<-(rc-.5)*2

}

## at this point rc represents an index of dissimilarity- multiply by -1 to convert to a similarity as specified in the original 1979 Raup Crick paper

if(report_similarity & !classic_metric){

rc<- rc*-1

}

## the switch to similarity is done differently if the original 0 to 1 range of the metric is used:

if(report_similarity & classic_metric){

rc<- 1-rc

}

##store the metric in the results matrix:

results[i,j]<-round(rc, digits=2)

}

}

if(as.distance.matrix){

results<-as.dist(results)

}

return(results)

}

#******************************END*******************************************

#=====================================================================

# 4. RC() function

#=====================================================================

#randomization test of Raup-Crick metric of Bray-Curtis index

#using Chase et al.'s code directly

#ssmat is a site-species matrix or table

RC<-function(ssmat,rtime)

{

obs=as.vector(raup_crick(ssmat,rtime=rtime))

return(obs)

}#

#******************************END*******************************************

#=====================================================================

# 5. sps.RC() function

#=====================================================================

#-------------------------------------------------------

# 5.1 old_sps.RC() function

#-------------------------------------------------------

#species' contributions to randomization test of

#Raup-Crick metric of Bray-Curtis index using Chase et al.'s code directly

#ssmat is a site-species matrix or table

sps.RC<-function(ssmat,rtime)

{

obs=as.vector(raup_crick(ssmat,rtime=rtime))

########################

#contribution of each species

rmat=matrix(0,ncol=length(obs),nrow=dim(ssmat)[2]+1)

rmat[1,]=obs

for(j in 1:dim(ssmat)[2])

{

ssmat2=ssmat[,-j]

tv=as.vector(raup_crick(ssmat2,rtime=rtime))

###################################################

rmat[j+1,]=tv

cat(j,"sps.RC finished...","\n")

}#j

########################

rownames(rmat)=c("OriginalBC",colnames(ssmat))

###################################################

return(rmat)

}#

#******************************END**********************************************

#=====================================================================

# 6. sps.RC.grouping() function

#=====================================================================

#-------------------------------------------------------

# 6.1 old_sps.RC.grouping() function

#-------------------------------------------------------

#species' or groups' contributions to randomization test of

#Raup-Crick metric of Bray-Curtis index using Chase et al.'s code directly

#ssmat is a site-species matrix or table

#groups is a list of vectors, as.list()

# # sps.RC.grouping<-function(ssmat,rtime,threads)

# sps.RC.grouping<-function(ssmat,rtime,groups)

# {

# #-------------------------

# #ready parallel run

# #-------------------------

# # threads <- makeCluster(threads)

# # registerDoParallel(threads)

#

# obs=as.vector(raup_crick(ssmat,rtime=rtime))

# ########################

# #contribution of each group

# rmat=matrix(0,ncol=length(obs),nrow=length(groups)+1)

# rmat[1,]=obs

#

# for(j in 1:length(groups))

# {

# IDS=which(colnames(ssmat)%in%groups[[j]]==TRUE)

# ssmat2=ssmat[,-IDS]

# tv=as.vector(raup_crick(ssmat2,rtime=rtime))

# ###################################################

# cat(j,"sps.RC.grouping finished...","\n")

# rmat[j+1,]=tv

# }#j

# rownames(rmat)=c("OriginalBC",paste("groups",1:length(groups),sep=""))

# ###################################################

#

# # #----------------------

# # # end parallel run

# # #----------------------

# # stopImplicitCluster()

# # stopCluster(threads)

#

# return(rmat)

# }#

#-------------------------------------------------------

# 6.2 new_sps.RC.grouping() function

#-------------------------------------------------------

#species' or groups' contributions to randomization test of

#Raup-Crick metric of Bray-Curtis index using Chase et al.'s code directly

#ssmat is a site-species matrix or table

#groups is a list of vectors, as.list()

# sps.RC.grouping<-function(ssmat,rtime,groups,threads)

sps.RC.grouping<-function(ssmat,rtime,groups){

#-------------------------

#ready parallel run

#-------------------------

# threads <- makeCluster(threads)

# clusterExport(threads,c("raup_crick","alpha_table_111"))

# registerDoParallel(threads)

obs=as.vector(raup_crick(ssmat,rtime=rtime))

########################

#contribution of each group

rmat=matrix(0,ncol=length(obs),nrow=length(groups)+1)

rmat[1,]=obs

#~~~~~~~~~~~~~~~~~~~~~~~~~~~~~~~~~~~~~~~~

if(!file.exists("Res_RC_group"))dir.create("Res_RC_group")

if(!file.exists("Res_RC_group/Inter_list"))dir.create("Res_RC_group/Inter_list")

foreach(j=1:length(groups),.packages = c("vegan","ape","picante","wrswoR"),

.export = c("raup_crick","alpha_table_111"))%dopar%{

IDS=which(colnames(ssmat)%in%groups[[j]]==TRUE)

ssmat2=ssmat[,-IDS]

tv=as.vector(raup_crick(ssmat2,rtime=rtime))

###########

cat(j,"sps.RC.grouping finished...","\n")

saveRDS(tv,paste0("./Res_RC_group/Inter_list/tv",j,".rds"))#批量导出

}

#~~~~~~~~~~~~~~~~~~~~~~~~~~~~~~~~~~~~~~~~

for (j in 1:length(groups)) {

tv <- readRDS(paste0("./Res_RC_group/Inter_list/tv",j,".rds"))

rmat[j+1,]=tv

cat(j,"sps.RC.grouping finished...","\n")

}#j

rownames(rmat)=c("OriginalBC",paste("groups",1:length(groups),sep=""))

#~~~~~~~~~~~~~~~~~~~~~~~~~~~~~~~~~~~~~~~~

# #----------------------

# # end parallel run

# #----------------------

# stopImplicitCluster()

# stopCluster(threads)

library(fs)

dir_delete("Res_RC_group")

return(rmat)

}

#******************************END**********************************************

#===============================================================================

# 7. beta.MNTD() function

#===============================================================================

#-------------------------------------------------------

# 7.2 beta.MNTD() function

#-------------------------------------------------------

# ssmat=ssmat;rtime=100;tree=tre

beta.MNTD<-function(ssmat,tree,rtime,abundance.weighted = FALSE, exclude.conspecifics = FALSE)

{

dis=cophenetic(tree)

tree1=tree

obs=as.vector(comdistnt(ssmat,dis,abundance.weighted))

rmat=vector()

#Parallel run

# threads <- makeCluster(threads)

# registerDoParallel(threads)

rmat <- foreach(i= 1:rtime,.combine = rbind,.packages = c("vegan","ape","picante")) %dopar% {

tree1$tip.label=sample(tree1$tip.label,length(tree1$tip.label),replace=FALSE)

dis1=cophenetic(tree1)

rv=as.vector(comdistnt(ssmat,dis1,abundance.weighted))

rmat=rbind(rmat,rv)

cat(i,"beta.MNTD finished...","\n")

return(rmat)#

}

nrow(rmat)

# rmat

# class(rmat)

##############

oneZ<-function(id)

{

z=(obs[id]-mean(rmat[,id]))/sd(rmat[,id])

return(z)

}#

##############

v =sapply(1:length(obs),oneZ)

##############

return(v)

# stopImplicitCluster()

# stopCluster(threads)

}

#******************************END**********************************************

#===============================================================================

# 8. sps.beta.MNTD() function

#===============================================================================

#-------------------------------------------------------

#contribution of each species in

#z score test of MNTD (mean nearest taxon distance)

#ssmat is a site-species matrix or table

#tree is the phylogeny, tip labels should match the species in ssmat!

# ssmat=ssmat; tree=tre; rtime=100; threads=10; groups=g; abundance.weighted = FALSE; exclude.conspecifics = FALSE

sps.beta.MNTD<-function(ssmat,tree,rtime,abundance.weighted = FALSE, exclude.conspecifics = FALSE)

{

#ready parellel run

# threads <- makeCluster(threads)

# registerDoParallel(threads)

dis=cophenetic(tree)

tree1=tree

obs=as.vector(comdistnt(ssmat,dis,abundance.weighted))

rmat=array(0,dim=c(rtime,length(obs),dim(ssmat)[2]+1))

#~~~~~~~~~~~~~~~~~~~~~~~~~~~~~~~~~~~~~~~~

if(!file.exists("Res_rmat"))dir.create("Res_rmat")

if(!file.exists("Res_rmat/Inter_list"))dir.create("Res_rmat/Inter_list")

foreach(i=1:rtime,.packages = c("vegan","ape","picante","wrswoR"))%dopar%{

tree1$tip.label=sample(tree1$tip.label,length(tree1$tip.label),replace=FALSE)

dis1=cophenetic(tree1)

rv=as.vector(comdistnt(ssmat,dis1,abundance.weighted))

saveRDS(rv,paste0("./Res_rmat/Inter_list/rv",i,".rds"))

saveRDS(dis1,paste0("./Res_rmat/Inter_list/dis",i,".rds"))

}

#~~~~~~~~~~~~~~~~~~~~~~~~~~~~~~~~~~~~~~~~

for (i in 1:rtime) {

dis1 <- readRDS(paste0("./Res_rmat/Inter_list/dis",i,".rds"))

#-------------------------------

#再次并行输出结果

#-------------------------------

foreach(j=1:dim(ssmat)[2],.packages = c("vegan","ape","picante","wrswoR"),.combine = "rbind")%dopar%{

ssmat2=ssmat[,-j]

id=which(rownames(dis1)==colnames(ssmat)[j])

dis2=dis1[-id,-id]

tv=as.vector(comdistnt(ssmat2,dis2,abundance.weighted))

saveRDS(tv,paste0("./Res_rmat/Inter_list/tv",i,"_",j,".rds"))

###################################################

# rmat[i,,j+1]=tv

}

#-------------------------------

cat(i,"rtime sps.beta.MNTD...","\n")

}

#~~~~~~~~~~~~~~~~~~~~~~~~~~~~~~~~~~~~~~~~

for (i in 1:rtime) {

rv <- readRDS(paste0("./Res_rmat/Inter_list/rv",i,".rds"))

dis1 <- readRDS(paste0("./Res_rmat/Inter_list/dis",i,".rds"))

rmat[i,,1] = rv

# cat(i,"rtime sps.beta.MNTD...","\n")

for(j in 1:dim(ssmat)[2]) {

tv <- readRDS(paste0("./Res_rmat/Inter_list/tv",i,"_",j,".rds"))

rmat[i,,j+1]=tv

}#j

}#i

###################################################

vmat=vector()

for(j in 1:(dim(ssmat)[2]+1))

{

oneZ<-function(id)

{

z=(obs[id]-mean(rmat[,id,j]))/sd(rmat[,id,j])

return(z)

}#

##############

v=sapply(1:length(obs),oneZ)

vmat=rbind(vmat,v)

}#j

rownames(vmat)=c("OriginalZ",colnames(ssmat))

###################################################

library(fs)

dir_delete("Res_rmat")

# stopImplicitCluster()

# stopCluster(threads)

return(vmat)

}#

#******************************END**********************************************

#===============================================================================

# 9. sps.beta.MNTD.grouping() function

#===============================================================================

#-------------------------------------------------------

# 9.2 sps.beta.MNTD.grouping() function

#-------------------------------------------------------

#contribution of given OTUs or groups of OTUs in

#z score test of MNTD (mean nearest taxon distance)

#ssmat is a site-species matrix or table

#tree is the phylogeny, tip labels should match the species in ssmat!

#groups is a list for which element is a vector showing OTU IDs

#as.list(c(1,2,3)) is good!

#=====================================================

# ssmat=ssmat; tree=tre; rtime=100; threads=10; groups=g; abundance.weighted = FALSE; exclude.conspecifics = FALSE

sps.beta.MNTD.grouping<-function(ssmat,tree,rtime,groups,abundance.weighted = FALSE, exclude.conspecifics = FALSE)

{

#reay parallel run

# threads <- makeCluster(threads)

# registerDoParallel(threads)

dis=cophenetic(tree)

tree1=tree

obs=as.vector(comdistnt(ssmat,dis,abundance.weighted))

rmat=array(0,dim=c(rtime,length(obs),dim(ssmat)[2]+1))

#~~~~~~~~~~~~~~~~~~~~~~~~~~~~~~~~~~~~~~~~

if(!file.exists("Res_rmat_group"))dir.create("Res_rmat_group")

if(!file.exists("Res_rmat_group/Inter_list"))dir.create("Res_rmat_group/Inter_list")

foreach(i=1:rtime,.packages = c("vegan","ape","picante","wrswoR"))%dopar%{

tree1$tip.label=sample(tree1$tip.label,length(tree1$tip.label),replace=FALSE)

dis1=cophenetic(tree1)

rv=as.vector(comdistnt(ssmat,dis1,abundance.weighted))

saveRDS(rv,paste0("./Res_rmat_group/Inter_list/rv",i,".rds"))#

saveRDS(dis1,paste0("./Res_rmat_group/Inter_list/dist",i,".rds"))

#rmat=rbind(rmat,rv)

}

#~~~~~~~~~~~~~~~~~~~~~~~~~~~~~~~~~~~~~~~~

#~~~~~~~~~~~~~~~~~~~~~~~~~~~~~~~~~~~~~~~~

for (i in 1:rtime) {

dis1 <- readRDS(paste0("./Res_rmat_group/Inter_list/dist",i,".rds"))

#-------------------------------

#再次并行输出结果

#-------------------------------

foreach(j=1:length(groups),.packages = c("vegan","ape","picante","wrswoR"),.combine = "rbind")%dopar%{

IDS=which(colnames(ssmat)%in%groups[[j]]==TRUE)

ssmat2=ssmat[,-IDS]

id=which(rownames(dis1)%in%colnames(ssmat)[IDS]==TRUE)

dis2=dis1[-id,-id]

tv=as.vector(comdistnt(ssmat2,dis2,abundance.weighted))

saveRDS(tv,paste0("./Res_rmat_group/Inter_list/tv",i,"_",j,".rds"))

###################################################

# rmat[i,,j+1]=tv

}

#-------------------------------

cat(i,"rtime sps.beta.MNTD.grouping...","\n")

}

#~~~~~~~~~~~~~~~~~~~~~~~~~~~~~~~~~~~~~~~~

for (i in 1:rtime) {

rv <- readRDS(paste0("./Res_rmat_group/Inter_list/rv",i,".rds"))

dis1 <- readRDS(paste0("./Res_rmat_group/Inter_list/dist",i,".rds"))

rmat[i,,1] = rv

cat(i,"rtime sps.beta.MNTD.grouping...","\n")

for(j in 1:length(groups)) {

tv <- readRDS(paste0("./Res_rmat_group/Inter_list/tv",i,"_",j,".rds"))

rmat[i,,j+1]=tv

}#j

}#i

###################################################

vmat=vector()

for(j in 1:(length(groups)+1))

{

oneZ<-function(id)

{

z=(obs[id]-mean(rmat[,id,j]))/sd(rmat[,id,j])

return(z)

}#

##############

v=sapply(1:length(obs),oneZ)

vmat=rbind(vmat,v)

}#j

rownames(vmat)=c("OriginalZ",paste("group",1:length(groups),sep=""))

###################################################

library(fs)

dir_delete("Res_rmat_group")

return(vmat)

}#

#******************************END**********************************************

#===============================================================================

# 10. all.in.one.grouping.Forward2 function

#===============================================================================

#-------------------------------------------------------

# 10.1 New_all.in.one.grouping.Forward2()

#-------------------------------------------------------

all.in.one.grouping.Forward2<-function(ssmat,tree,rtime,threads=1,site.groups=NULL,sps.groups=NULL,sps.contribution=FALSE,abundance.weighted=FALSE,exclude.conspecifics=FALSE)

{

#-------------------------

#ready parallel run

#-------------------------

threads <- makeCluster(threads)

clusterExport(threads,c("raup_crick","alpha_table_111"))

registerDoParallel(threads)

sn=rownames(ssmat)

if(is.null(sn))

{

sn=1:dim(ssmat)[1]

}

SNP=pair.ID(sn)

#

#

if(sps.contribution==FALSE)

{

res1=beta.MNTD(ssmat,tree,rtime,abundance.weighted,exclude.conspecifics)

#res2=RC(ssmat,tree,rtime,threads)

res2=RC(ssmat,rtime)

}else

{

if(is.null(sps.groups))

{

res1=sps.beta.MNTD(ssmat,tree,rtime,abundance.weighted,exclude.conspecifics)

res2=sps.RC(ssmat,tree,rtime)

}else

{

res2=sps.RC.grouping(ssmat,rtime,sps.groups)

write.csv(res2,"sps.RC.grouping.csv",row.names = T)

res1=sps.beta.MNTD.grouping(ssmat,tree,rtime,sps.groups,abundance.weighted,exclude.conspecifics)

write.csv(res1,"sps.beta.MNTD.grouping.csv",row.names = T)

}

}#

############################

if(sps.contribution==FALSE)

{

ids1=which(res1>=2)

ids2=which(res1<= -2)

ids3=which(-2< res1 & res1<2 & res2>=.95)

ids4=which(-2< res1 & res1<2 & res2<= -.95)

ids5=which(-2< res1 & res1<2 & res2> -.95 & res2< .95)

############################

out1=c(length(ids1),

length(ids2),

length(ids3),

length(ids4),

length(ids5))

out1=out1/sum(out1)

names(out1)=c("var.selection","hom.selection","dispersal.lim","hom.dispersal","drift")

########################################################

#contribution of each site or each site group

if(is.null(site.groups))

{

out2=vector()

for(i in 1:length(sn))

{

ids=which(SNP[,1]==sn[i] | SNP[,2]==sn[i])

ids1=which(res1[ids]>=2)

ids2=which(res1[ids]<= -2)

ids3=which(-2< res1[ids] & res1[ids]<2 & res2[ids]>=.95)

ids4=which(-2< res1[ids] & res1[ids]<2 & res2[ids]<= -.95)

ids5=which(-2< res1[ids] & res1[ids]<2 & res2[ids]> -.95 & res2[ids]< .95)

############################

ww=c(length(ids1),

length(ids2),

length(ids3),

length(ids4),

length(ids5))

ww=ww/sum(ww)

out2=rbind(out2,ww)

}#i

colnames(out2)=c("var.selection","hom.selection","dispersal.lim","hom.dispersal","drift")

rownames(out2)=sn

}else

{

out2=vector()

for(i in 1:length(site.groups))

{

ids=which(SNP[,1]%in%site.groups[[i]]==TRUE | SNP[,2]%in%site.groups[[i]]==TRUE)

ids1=which(res1[ids]>=2)

ids2=which(res1[ids]<= -2)

ids3=which(-2< res1[ids] & res1[ids]<2 & res2[ids]>=.95)

ids4=which(-2< res1[ids] & res1[ids]<2 & res2[ids]<= -.95)

ids5=which(-2< res1[ids] & res1[ids]<2 & res2[ids]> -.95 & res2[ids]< .95)

############################

ww=c(length(ids1),

length(ids2),

length(ids3),

length(ids4),

length(ids5))

ww=ww/sum(ww)

out2=rbind(out2,ww)

}#i

colnames(out2)=c("var.selection","hom.selection","dispersal.lim","hom.dispersal","drift")

rownames(out2)=paste("site.groups",1:length(site.groups),sep="")

}

########################################################

#

return(list(beta.partition=out1,site.contribution=out2))

}else

{

ids1=which(res1[1,]>=2)

ids2=which(res1[1,]<= -2)

ids3=which(-2< res1[1,] & res1[1,]<2 & res2[1,]>=.95)

ids4=which(-2< res1[1,] & res1[1,]<2 & res2[1,]<= -.95)

ids5=which(-2< res1[1,] & res1[1,]<2 & res2[1,]> -.95 & res2[1,]< .95)

############

out1=c(length(ids1),

length(ids2),

length(ids3),

length(ids4),

length(ids5))

out1=out1/sum(out1)

names(out1)=c("var.selection","hom.selection","dispersal.lim","hom.dispersal","drift")

########################################################

#contribution of each site or each site group

if(is.null(site.groups))

{

out2=vector()

for(i in 1:length(sn))

{

ids=which(SNP[,1]==sn[i] | SNP[,2]==sn[i])

ids1=which(res1[1,ids]>=2)

ids2=which(res1[1,ids]<= -2)

ids3=which(-2< res1[1,ids] & res1[1,ids]<2 & res2[1,ids]>=.95)

ids4=which(-2< res1[1,ids] & res1[1,ids]<2 & res2[1,ids]<= -.95)

ids5=which(-2< res1[1,ids] & res1[1,ids]<2 & res2[1,ids]> -.95 & res2[1,ids]< .95)

############################

ww=c(length(ids1),

length(ids2),

length(ids3),

length(ids4),

length(ids5))

ww=ww/sum(ww)

out2=rbind(out2,ww)

}#i

colnames(out2)=c("var.selection","hom.selection","dispersal.lim","hom.dispersal","drift")

rownames(out2)=sn

}else

{

out2=vector()

for(i in 1:length(site.groups))

{

ids=which(SNP[,1]%in%site.groups[[i]]==TRUE | SNP[,2]%in%site.groups[[i]]==TRUE)

ids1=which(res1[1,ids]>=2)

ids2=which(res1[1,ids]<= -2)

ids3=which(-2< res1[1,ids] & res1[1,ids]<2 & res2[1,ids]>=.95)

ids4=which(-2< res1[1,ids] & res1[1,ids]<2 & res2[1,ids]<= -.95)

ids5=which(-2< res1[1,ids] & res1[1,ids]<2 & res2[1,ids]> -.95 & res2[1,ids]< .95)

############################

ww=c(length(ids1),

length(ids2),

length(ids3),

length(ids4),

length(ids5))

ww=ww/sum(ww)

out2=rbind(out2,ww)

}#i

colnames(out2)=c("var.selection","hom.selection","dispersal.lim","hom.dispersal","drift")

rownames(out2)=paste("site.groups",1:length(site.groups),sep="")

}

########################################################

#

############

#species' contributions

sv=vector()

if(is.null(sps.groups))

{

IID=1:dim(ssmat)[2]

}else

{

IID=1:length(sps.groups)

}

##################

for(i in IID)

{

# if(out1[1]>0)

# {

# di1=which(res1[i+1,ids1]< -2) #significant hom.selection

# }

# if(out2[2]>0)

# {

# di2=which(res1[i+1,ids1]> 2) #significant var.selection

# }

##########

id1=which(-2< res1[1,] & res1[1,]<2 & res1[i+1,]< -2) #remarkable contribution to var.selection

id2=which(-2< res1[1,] & res1[1,]<2 & res1[i+1,]> 2) #remarkable contribution to hom.selection

#id1=which(-2< res1[1,] & res1[i+1,]< -2) #remarkable contribution to var.selection

#id2=which(res1[1,]<2 & res1[i+1,]> 2) #remarkable contribution to hom.selection

id3=which(-2>= res1[1,] & res1[i+1,]> -2) #remarkable contribution to hom.selection

id4=which(2<= res1[1,] & res1[i+1,]<2) #remarkable contribution to var.selection

##########

id5=which(-2< res1[1,] & res1[1,]<2 & -2< res1[i+1,] & res1[i+1,]<2 & res2[1,]> -.95 & res2[1,]< .95 & res2[i+1,]< -.95) #remarkable contribution to lim.dispersal

id6=which(-2< res1[1,] & res1[1,]<2 & -2< res1[i+1,] & res1[i+1,]<2 & res2[1,]> -.95 & res2[1,]< .95 & res2[i+1,]> .95) #remarkable contribution to hom.dispersal

id7=which(-2< res1[1,] & res1[1,]<2 & -2< res1[i+1,] & res1[i+1,]<2 & res2[1,]< -.95 & res2[i+1,]>= -.95) #remarkable contribution to hom.dispersal

id8=which(-2< res1[1,] & res1[1,]<2 & -2< res1[i+1,] & res1[i+1,]<2 & res2[1,]>= .95 & res2[i+1,]< .95) #remarkable contribution to lim.dispersal

id9=which(-2< res1[1,] & res1[1,]<2 & -2< res1[i+1,] & res1[i+1,]<2 & res2[1,]< .95 & res2[i+1,]> -.95)

##########

#vv=c(length(unique(c(id1,id4))),length(unique(c(id2,id3))),length(c(id5,id8)),length(c(id6,id7)),length(id9))

vv=c(length(id4),length(id3),length(id8),length(id7),length(id9))

#sv=rbind(sv,vv)

#2020-08-23:

sv=rbind(sv,vv/sum(vv))

}#i

#2020-08-23:

#sv=sv/dim(res1)[2]

#sv=cbind(sv,1-rowSums(sv))

#

if(is.null(sps.groups))

{

rownames(sv)=colnames(ssmat)

}else

{

rownames(sv)=paste("sps.groups",1:length(sps.groups),sep="")

}

colnames(sv)=c("var.selection","hom.selection","dispersal.lim","hom.dispersal","drift")

####

return(list(beta.partition=out1,site.contribution=out2,sps.contribution=sv))

}#

#----------------------

# end parallel run

#----------------------

stopImplicitCluster()

stopCluster(threads)

}#en

#*******************************************************************************

#-------------------------------------------------------------------------------

# Example data

#===============================================================================

#---------------------

# 1.otu table

#---------------------

ssmat=matrix(sample(0:1,100,replace=TRUE),ncol=10)

rownames(ssmat)=paste("site",1:10,sep="")

colnames(ssmat)=paste("otu",1:10,sep = "")

head(ssmat)

#---------------------

# 2.tree

#---------------------

tre=rtree(10)

tre$tip.label <- colnames(ssmat)

plot(tre)

dat=cbind(colnames(ssmat),c(1,1,1,2,2,2,3,3,3,3))

head(dat)

g=dat2group(dat)

g

sitedat=cbind(rownames(ssmat),c(1,1,1,2,2,3,3,4,4,4))

sitedat

sg=dat2group(sitedat)

sg

system.time(

ana2 <- all.in.one.grouping.Forward2(ssmat = ssmat,tree = tre,rtime =100,threads = 5,site.groups=sg,sps.groups=g,sps.contribution=TRUE,abundance.weighted=FALSE)

)

ana2
